# Supplementary material for: Multiple Glycation Sites in Blood Plasma Proteins as an Integrated Biomarker of Type 2 Diabetes Mellitus
Source: Int J Mol Sci. 2019 May 10;20(9):2329. doi: 10.3390/ijms20092329 (PMC6539793; doi:10.3390/ijms20092329)
Supplement: Supplementary file 1 [file ijms-20-02329-s001.pdf]

Multiple glycation sites in blood plasma proteins as an integrated biomarker of type 2 diabetes mellitus

Supplementary Material

Alena Soboleva,[1,2] Gregory Mavropulo-Stolyarenko,[1] Tatiana Karonova,[3,4] Domenika Thieme,[5] Wolfgang Hoehenwarter,[5] Christian Ihling,[6] Vasily Stefanov,[1] Tatiana Grishina,[1] Andrej Frolov[2*]

[1]Department of Biochemistry, St. Petersburg State University, [2]Department of Bioorganic Chemistry, Leibniz Institute of Plant Biochemistry, [3]Federal Almazov North-West Medical Research Centre, [4]The First Pavlov St. Petersburg State Medical University, [5]Proteome Analytics Research Group, Leibniz Institute of Plant Biochemistry and [6]Institute of Pharmacy, Martin Luther University of Halle-Wittenberg

*Corresponding author:

Dr. Andrej Frolov

Leibniz Institute of Plant Biochemistry

Department of Bioorganic Chemistry

Weinberg 3, 06120

Halle/Saale, Germany

Tel. +49(0)34555821350

Fax. +49(0)34555821309

Directory

Tables.. 4

Table S1 Protein concentrations and average total signal intensities calculated for individual samples (lanes) separated by SDS-PAGE.. 4

Table S2 Validation of the label-free quantification method with intra-day and inter-day precision values determined for individual glycated peptides 6

Table S3 Identification of glycated peptides, up-regulated in T2DM patients 7

Table S4 Values of the first 9 principal components, obtained by PCA......................... 9

Table S5 Occurrence of variable sets in 1000 runs of the variance inflation factor-based optimization procedure with cutoff value set to 10 11

Table S6 Occurrence of variable sets in 1000 runs of the variance inflation factor based optimization procedure with cutoff value set to 5 12

Table S7 Ion source and mass analyzer settings applied for QqTOF-MS experiments 13

Table S8 Instrument settings used for LIT-Orbitrap-MS and -MS/MS experiments.. 14

Figures 16

Figure S1 SDS-PAGE electropherograms of individual plasma protein samples....... 16

Figure S2 Example of a SDS-PAGE electrophoregram representing individual plasma protein tryptic digests. 17

Figure S3 Integral peak areas observed for the signals of the peptides, up-regulated in tryptic plasma digests obtained from T2DM patients. 18

Figure S4 Tandem mass spectra of differentially glycated peptides identified by Mascot database search and manual interpretation.. 21

Figure S5 Tandem mass spectra of differentially glycated peptides identified by manual interpretation of the MS/MS data 40

Figure S6 Pearson's cross-correlation coefficient plot for variables 44

Tables

Table S1 Protein concentrations and average densities calculated for individual samples (lanes) separated by SDS-PAGE

Sample	Concentration (mg/mL)	Density (AU)
Control-1	82.4	17999
Control-2	82.3	18392
Control-3	58.1	19867
Control-4	45.3	19966
Control-5	52.5	19966
Control-6	36.2	20350
Control-7	58.0	20254
Control-8	58.7	18814
Control-9	67.9	18718
Control-10	67.1	19966
Control-11	58.5	19678
Control-12	74.9	18408
Control-13	54.3	18408
Control-14	37.1	20567
Control-15	70.1	18535
Control-16	68.8	16377
Control-17	69.5	17647
Control-18	72.5	20313
T2DM-1	42.7	19700

T2DM-2	58.6	18600
T2DM-3	56.0	19200
T2DM-4	64.5	17400
T2DM-5	57.8	18100
T2DM-6	75.5	18100
T2DM-7	78.5	17200
T2DM-8	76.1	18800
T2DM-9	52.2	20200
T2DM-10	62.4	19801
T2DM-11	51.5	19668
T2DM-12	55.4	20200
T2DM-13	47.5	18738
T2DM-14	48.1	20599
T2DM-15	62.8	20200
T2DM-16	59.9	21795
T2DM-17	52.4	22326
T2DM-18	60.3	20753
T2DM-19	65.7	20359
T2DM-20	48.6	20163

Table S2 Validation of the label-free quantification method with intraday and inter-day precision values determined for individual glycated peptides

**Peptide**[a]	*Intraday precision (n = 3)*		*Inter-day precision (n = 3/day)*[b]	
	**t_R (min) ± SD (RSD%)**	**S_a (counts) ± SD (RSD%)**	**t_R (min) ± SD (RSD%)**	**S_a (counts) ± SD (RSD%)**
5	25.8 ± 0.07 (0.27)	1.68E+06 ± 5.90E+04 (3.51)	25.5 ± 0.24 (0.94)	1.59E+06 ± 1.15E+05 (7.25)
6	26.4 ± 0.14 (0.54)	5.20E+05 ± 1.74E+04 (3.35)	26.2 ± 0.20 (0.78)	5.86E+05 ± 6.15E+04 (10.50)
9	31.0 ± 0.10 (0.34)	1.73E+06 ± 7.37E+04 (4.27)	31.0 ± 0.12 (0.37)	1.90E+06 ± 1.62E+05 (8.57)
11	32.0 ± 0.07 (0.22)	6.13E+05 ± 1.56E+04 (2.55)	31.9 ± 0.11 (0.36)	6.52E+05 ± 4.29E+04 (6.59)
12	33.1 ± 0.06 (0.17)	6.74E+04 ± 7.42E+03 (11.01)	33.1 ± 0.07 (0.21)	6.64E+04 ± 1.09E+04 (16.46)
14	36.1 ± 0.04 (0.10)	1.87E+06 ± 2.08E+04 (1.11)	36.0 ± 0.09 (0.24)	1.80E+06 ± 9.92E+04 (5.52)
16	37.4 ± 0.07 (0.19)	2.75E+06 ± 3.20E+04 (1.17)	37.4 ± 0.10 (0.26)	2.61E+06 ± 1.81E+05 (6.92)
17	37.5 ± 0.06 (0.15)	2.05E+05 ± 9.57E+03 (4.68)	37.5 ± 0.05 (0.14)	2.29E+05 ± 3.60E+04 (15.67)
25	45.8 ± 0.04 (0.08)	1.82E+06 ± 5.36E+04 (2.94)	45.8 ± 0.05 (0.10)	1.88E+06 ± 1.84E+05 (9.77)
36	25.7 ± 0.06 (0.22)	5.63E+04 ± 6.63E+02 (1.18)	25.7 ± 0.12 (0.47)	6.33E+04 ± 5.45E+03 (8.61)

Validation was performed with a pooled T2DM plasma; [a] peptides labeled as in Table S6; [b]an inter-day precision; was determined on three consecutive days; SD, standard deviation; RSD%, relative standard deviation percentage.

Table S3 Identification of glycated peptides, up-regulated in T2DM patients, by Mascot database search with Proteome Discoverer software

Nr	Sequence	m/z_{obs}	z	Δm (ppm)	Ion score	Expectation value
			**Human serum albumin**			
1	VTK*C*C*TESLVNR	543.5934	3	-1.3	28	2.64E-02
2	RYK*AAFTEC*C*QAADK	660.9643	3	1.1	34	7.87E-03
3	LK*EC*C*EKPLLEK	570.2864	3	3.9	manual interpretation	
4	C*ASLQK*FGER	453.2184	3	1.8	manual interpretation	
5	LAK*TYETTLEK	729.8835	2	-1.0	30	2.07E-02
6	YK*AAFTEC*C*QAADK	608.9324	3	-1.5	45	1.30E-04
7	ETYGEMADC*C*AK*QEPER[a]	745.9667	3	0	23	9.66E-02
8	YIC*ENQDSISSK*LK	616.2968	3	0	14	8.17E-01
9	FK*DLGEENFK	463.5584	3	-0.9	17	3.32E-01
10	TYETTLEK*C*C*AAADPHEC*YAK[a]	670.7869	4	-0.9	10	2.49E-01
11	K*YLYEIAR	406.5519	3	1.2	29	2.58E-02
12	K*QTALVELVK	430.9228	3	0.7	56	3.93E-05
13	K*VPQVSTPTLVEVSR	901.4979	2	1.7	53	1.08E-04
14	ADLAK*YIC*ENQDSISSK	701.9975	3	0.7	46	4.23E-04
15	LVTDLTK*VHTEC*C*HGDLLEC*ADDR	1007.1184	3	1.7	13	1.03
16	TC*VADESAENC*DK*SLHTLFGDK	665.5443	4	-1.4	16	0.24
17	K*LVAASQAALGL	652.3774	2	0.2	32	9.22E-03
18	AAFTEC*C*QAADK*AAC*LLPK	1144.0238	2	0.7	14	0.73
19	LVNEVTEFAK*TC*VADESAENC*DK	931.0820	3	0.5	58	1.47E-05
20	AAC*LLPK*LDELR	780.9185	2	0.9	11	2.29
21	SLHTLFGDK*LC*TVATLR	698.7045	3	3.6	27	4.02E-02
22	EFNAETFTFHADIC*TLSEK*ER	903.0778	3	0.7	39	2.64E-03
23	TC*VADESAENC*DK*SLHTLFGDKLC*TVATLR[a]	1191.8881	3	0.7	20	2.23E-01
24	EQLK*AVM*DDFAAFVEK	673.6594	3	0.3	50	2.54E-04
25	VFDEFK*PLVEEPQNLIK	736.3884	3	-0.4	42	1.84E-03
26	AEFAEVSK*LVTDLTK	906.9786	2	-0.7	33	1.49E-02
27	RHPYFYAPELLFFAK*	687.6876	3	0.6	20	0.19
			**Alpha-2-macroglobulin**			
28	LVDGK*GVPIPNK[a]	466.9322	3	2.4	20	0.21
29	ALLAYAFALAGNQDK*R[a]	628.6669	3	0.8	35	6.68E-03
			**Apolipoprotein A1**			
30	LAEYHAK*ATEHLSTLSEK	730.7012	3	2.2	12	1.40

**Ceruloplasmin precursor**						
31	VTFHNK*GAYPLSIEPIGVR	754.0687	3	3.2	36	4.49E-03
**Complement C4-A**						
32	RC*SVFYGAPSK*SR	838.9064	2	1.0	manual interpretation	
**FLJ00385 protein**						
33	VSNK*ALPAPIEK	476.9365	3	0.6	16	0.41
**Ig kappa chain C region**						
34	VQWK*VDNALQSGNSQESVTEQDSK[a]	710.5862	7	0.7	manual interpretation	
35	DSTYSLSSTLTLSK*ADYEK	757.6974	3	0	18	0.30
**Serotransferrin**						
36	GDVAFVK*HQTVPQNTGGK	512.0131	4	0.6	27	3.61E-02
37	DDTVC*LAK*LHDR	535.5911	3	0.7	manual interpretation	
38	K*PVDEYKDC*HLAQVPSHTVVAR[a]	678.5904	4	2.9	26	4.77E-02
39	SK*EFQLFSSPHGK	551.9417	3	1.6	30	2.07E-02
40	DLLFK*DSAHGFLK	826.9306	2	1.3	manual interpretation	
41	DGAGDVAFVK*HSTIFENLANK	799.0640	3	0.6	57	4.33E-05
**Vitamin D-binding protein precursor**						
42	TSALSAK*SC*ESNSPFPVHPGTAEC* C*TK	1029.1237	3	-0.1	31	1.77E-02

Tandem mass spectra were searched against a FASTA fail containing sequences of the proteins annotated by on MS level. The search relied on Mascot engine within Proteome Discoverer 1.4 software (Thermo-Fisher Scientific, Bremen, Germany) with the following settings: peptide tolerance – 7 ppm, MS/MS match tolerance – 0.8 Da, 3 missed cleavage sites per peptide. C*, M*, and K* denote carbamidomethylated cysteine, methione sulfoxide, and fructosamine lysine residues; [a]denote peptides, not demonstrating characteristic patterns of water and formaldehyde losses from the quasi-molecular ions.

Table S4 Values of the first 9 principal components calculated for individual samples

Sample	PC1	PC2	PC3	PC4	PC5	PC6	PC7	PC8	PC9
Control-1	-6.90927	-3.77376	3.366382	-1.70322	-3.81814	2.176394	-0.17328	0.66696	-0.0402
Control-2	-9.72634	0.008476	-0.32452	1.97793	2.001214	3.653437	0.406535	0.43305	0.487818
Control-3	-10.7529	-1.86911	0.755203	4.125013	0.035391	-1.3975	1.419009	-0.17474	-0.44569
Control-4	-12.0235	-0.77665	-2.66447	-1.60283	0.81114	-0.37982	-0.07854	1.476437	-1.74208
Control-5	-5.45327	-0.00908	1.092913	-1.22833	1.285674	-0.01509	1.715587	-0.59492	-0.04299
Control-6	4.865353	-3.07951	-1.25929	-0.26811	-0.77428	-0.73139	-0.31996	0.607199	0.657199
Control-7	-3.12423	-2.05148	-0.137	0.579639	0.35365	-0.72019	0.794758	-0.16966	-0.13167
Control-8	-1.1587	-3.52968	-0.35886	-0.27253	0.675693	-1.20292	-0.01191	0.87213	-0.03061
Control-9	-9.53396	-0.34967	-2.35503	0.540386	-1.2818	-0.53632	-2.10214	-1.58089	0.273661
Control-10	-7.54426	0.567921	-2.21158	-3.42791	0.061783	0.504106	0.809411	-1.94496	0.774222
Control-11	-4.21882	0.217882	-0.62156	1.529056	-0.27362	-0.08434	-0.91317	-0.5062	0.24725
Control-12	-7.22376	3.2589	0.137456	0.39575	-0.2449	-0.71811	-1.03368	1.228371	1.44945
Control-13	-2.81688	2.246749	0.912564	-0.27797	0.259397	0.179775	0.543872	0.008089	0.748985
Control-14	-4.43971	-0.29771	4.240926	-2.13072	1.177645	-2.52559	-0.29342	-0.549	0.335483
Control-15	-2.0579	1.712797	-0.12566	-0.9078	0.360628	-0.4092	0.461762	1.374264	0.554485
Control-16	2.734639	0.369317	0.740999	-0.21157	0.040202	0.12835	0.95466	-0.29977	-0.1517
Control-17	1.381772	2.537375	-0.43162	0.259299	-1.02821	-0.3799	0.639254	0.784523	-0.37434
Control-18	3.503061	0.859472	0.493034	0.857494	-0.61865	0.030769	0.152137	-0.61703	-0.20201
T2DM-1	1.725534	2.131738	0.279371	1.485107	-1.34802	-0.10314	0.027513	-0.42971	-0.41295
T2DM-2	-1.12184	3.922795	-1.11655	-0.77376	-1.92767	-0.57406	0.855911	0.17752	-1.33988
T2DM-3	6.226949	-0.11737	-0.38769	-0.75826	0.041503	0.533784	0.559322	0.218825	-0.31003
T2DM-4	6.31563	0.117719	0.031493	-0.8306	0.486114	0.570777	0.505946	-0.03789	0.187641

T2DM-5	4.761694	0.249486	-0.27386	0.235983	-0.05551	0.176459	0.715513	-0.23558	-0.27713
T2DM-6	4.58551	0.45059	-0.02154	0.759595	-0.44398	0.216132	0.193883	-0.32081	-0.32897
T2DM-7	4.263196	0.781782	0.637191	0.021753	-0.20645	0.201171	0.207269	-0.74278	-0.1945
T2DM-8	4.191279	1.80896	1.151126	0.634908	-0.04732	-0.09009	-0.40673	0.985693	0.4492
T2DM-9	5.164144	-0.41158	0.340448	1.097828	-0.09422	0.057176	0.168759	-0.08542	0.039768
T2DM-10	5.37047	-0.17063	0.435073	-0.23356	0.460375	0.505244	0.62867	-0.25727	0.227624
T2DM-11	2.936374	0.260893	0.126958	-0.65556	1.214457	0.733857	-1.39367	-0.15199	-1.91814
T2DM-12	3.922547	-0.95657	-0.79157	0.580972	0.05942	-0.09234	-0.08845	-0.16581	0.11396
T2DM-13	1.884675	0.064054	-0.47285	0.515191	0.270499	0.212301	-0.07663	-0.29935	1.149344
T2DM-14	5.336663	-1.65181	-0.94204	0.077425	0.392258	0.001881	-0.28935	0.234507	0.446973
T2DM-15	2.001702	-0.79855	0.415646	1.529702	-0.11419	-0.41084	-0.32363	-0.54786	-0.33178
T2DM-16	-1.01984	1.333863	2.742121	-0.07774	1.429707	0.638835	-2.39127	-0.33944	-0.86005
T2DM-17	1.641237	0.904341	-0.98187	-0.57583	-0.01739	-0.02861	-0.85906	0.783727	0.90269
T2DM-18	5.893631	-1.79175	-0.48891	-0.29707	0.448484	-0.47676	-0.2439	0.533485	-0.02198
T2DM-19	5.154185	-1.32289	-1.59433	-0.39914	-0.20536	-0.13276	-0.47644	-0.29631	0.182085
T2DM-20	5.264906	-0.8473	-0.33809	-0.5705	0.634467	0.488513	-0.28454	-0.03737	-0.07112

Table S5 Occurrence of variable sets in 1000 runs of the variance inflation factor-based optimization procedure with cutoff value set to 10

Variable set	Count
**V10 V12 V28 V29 V30 V32 V33 V34 V39 V40 V41 V42 V6 V7 V9**	444
V10 V12 V28 V29 V30 V32 V33 V34 V38 V39 V40 V41 V42 V6 V7	143
V10 V12 V28 V29 V30 V32 V33 V34 V39 V41 V42 V6 V7 V9	107
V10 V12 V26 V28 V29 V30 V32 V33 V34 V35 V38 V39 V40 V41 V42 V7	89
V10 V12 V26 V28 V29 V30 V32 V33 V34 V35 V39 V40 V41 V42 V7	52
V10 V12 V13 V28 V29 V30 V33 V34 V38 V39 V40 V41 V42 V6 V7	44
V10 V12 V28 V29 V30 V32 V33 V34 V39 V40 V41 V42 V6 V7	24
V10 V12 V28 V29 V30 V32 V33 V34 V35 V39 V41 V42 V7 V9	23
V10 V12 V28 V29 V30 V32 V33 V34 V35 V39 V40 V41 V42 V7 V9	12
V10 V12 V13 V28 V29 V30 V33 V34 V39 V40 V41 V42 V6 V7 V9	8
V10 V12 V26 V28 V29 V30 V32 V33 V34 V39 V40 V41 V42 V7	8
V10 V12 V26 V28 V29 V30 V32 V33 V34 V39 V41 V42 V7 V9	7
V10 V12 V26 V28 V29 V30 V32 V33 V34 V38 V39 V40 V41 V42 V7	6
V10 V12 V28 V29 V30 V32 V33 V34 V39 V41 V42 V6 V7 V8	6
V10 V12 V13 V26 V28 V29 V30 V33 V34 V35 V38 V39 V40 V41 V42 V7	3
V10 V12 V28 V29 V30 V32 V33 V34 V35 V38 V39 V40 V41 V42 V7	3
V10 V12 V13 V28 V29 V30 V33 V34 V39 V40 V41 V42 V6 V7	2
V10 V12 V13 V28 V29 V30 V34 V35 V38 V39 V40 V41 V42 V6 V7	2
V10 V12 V26 V28 V29 V30 V32 V33 V34 V35 V38 V39 V41 V42 V7	2
V10 V12 V26 V28 V29 V30 V32 V34 V35 V38 V39 V40 V41 V42 V7	2
V10 V24 V28 V29 V30 V32 V33 V34 V39 V41 V42 V6 V7 V9	2
V10 V11 V12 V29 V30 V32 V33 V34 V39 V41 V42 V6 V7 V9	1
V10 V12 V13 V26 V28 V29 V30 V31 V33 V34 V39 V40 V41 V42 V7	1
V10 V12 V13 V26 V28 V29 V30 V33 V34 V38 V39 V40 V41 V42 V7	1
V10 V12 V13 V28 V29 V30 V31 V34 V38 V39 V40 V41 V42 V7	1
V10 V12 V26 V28 V29 V30 V32 V33 V34 V35 V39 V41 V42 V7	1
V10 V12 V26 V28 V29 V30 V32 V33 V34 V38 V39 V41 V42 V7	1
V10 V12 V26 V29 V30 V32 V33 V34 V39 V4 V40 V41 V42 V7 V9	1
V10 V12 V28 V29 V30 V32 V33 V34 V39 V41 V42 V7 V9	1
V10 V12 V28 V29 V30 V32 V34 V35 V39 V40 V41 V42 V6 V7 V9	1
V10 V12 V28 V29 V30 V32 V34 V35 V39 V41 V42 V6 V7 V9	1
V10 V24 V28 V29 V30 V32 V33 V34 V39 V40 V41 V42 V6 V7 V9	1

The variables are numbered according Tables 1 and S3.

Table S6 Occurrence of variable sets in 1000 runs of the variance inflation factor based optimization procedure with cutoff value set to 5

Variable set	Count
**V10 V12 V28 V29 V30 V33 V34 V39 V41 V42 V6**	404
**V10 V12 V28 V29 V30 V32 V33 V34 V39 V41 V42 V7**	306
V10 V12 V28 V29 V30 V32 V34 V35 V39 V41 V42 V7	99
V10 V12 V28 V29 V30 V33 V34 V39 V41 V42 V7	62
V10 V12 V28 V29 V30 V34 V35 V39 V41 V42 V6	32
V10 V12 V28 V29 V30 V34 V35 V39 V41 V42 V7	32
V10 V12 V28 V29 V30 V33 V34 V39 V42 V6	13
V10 V12 V28 V29 V30 V32 V33 V34 V39 V42 V7	11
V10 V12 V28 V29 V30 V32 V33 V34 V39 V41 V42	10
V10 V12 V28 V29 V30 V34 V39 V41 V42 V6	5
V10 V12 V28 V29 V30 V32 V34 V35 V39 V41 V42	4
V10 V12 V28 V29 V30 V34 V39 V41 V42 V6 V9	4
V12 V28 V29 V30 V31 V32 V34 V39 V41 V42 V7 V9	4
V10 V12 V28 V29 V30 V31 V34 V39 V41 V42 V7	2
V10 V12 V28 V29 V30 V34 V39 V41 V42 V7	2
V10 V11 V12 V29 V30 V33 V34 V39 V41 V42 V6	1
V10 V12 V26 V28 V29 V32 V33 V34 V39 V41 V42 V7	1
V10 V12 V28 V29 V30 V32 V33 V34 V41 V42 V7	1
V10 V12 V28 V29 V30 V32 V34 V39 V41 V42 V7	1
V10 V12 V28 V29 V30 V32 V34 V39 V41 V42 V7 V9	1
V10 V12 V28 V29 V30 V34 V39 V41 V42 V7 V9	1
V10 V12 V29 V30 V32 V33 V34 V39 V42 V7 V9	1
V10 V28 V29 V30 V32 V33 V34 V39 V41 V42 V7	1
V12 V28 V29 V30 V32 V34 V35 V39 V41 V42 V7 V9	1
V12 V29 V30 V31 V32 V34 V39 V41 V42 V7 V9	1

The variables are numbered according Tables 1 and S3.

Table S7 Ion source and mass analyzer settings applied for QqTOF-MS experiments

**Parameter**	**Settings**
MS Conditions	
Ionization mode	Positive
Ion spray voltage	3.5 kV
Nebulizer	30 psig
Capillary temperature	300 °C
Gas flow	5 L/min
Front mirror voltage	7000 V
Mid mirror voltage	1669.2 V
Back mirror voltage	1250 V
Mass to charge ratio (*m*/*z*) range	400 – 2000
Mass resolution	36000-39000
Scan rate/frequency	3 Hz

Table S8 Instrument settings used in LIT-Orbitrap-MS and -MS/MS experiments

Parameter	Setting
MS conditions	
Ionization mode	Positive
Resolution	30.000
Ion spray voltage (IS)	1900 V
Capillary temperature	275 °C
Mass to charge ratio (*m*/*z*) range	400 – 1850
MS/MS conditions	
Fragmentation	Collision activated dissociation
Isolation width	2 Da
Charge state rejected	1+
Normalized collision energy	35
Activation frequency	0.25
Activation time	10 ms
Parent mass width	± 0.5 Da
Reject mass width	± 10 ppm
Dynamic exclusion repeat count	1
Dynamic exclusion repeat duration	30 s

Dynamic exclusion duration	90 s
Dynamic exclusion mass width	± 10 ppm
Database search settings	
Analysis program	Proteome Discoverer
Search engine	Mascot
Protease	Trypsin
Missed cleavage sites	3
Modification	Mass increment (Da) / amino acids
Carbamidomethyl (cam)	+57.0215 / C
Oxidation (ox)	+15.9949 / M
Amadori (Am)	+162.0528/ K

Figures

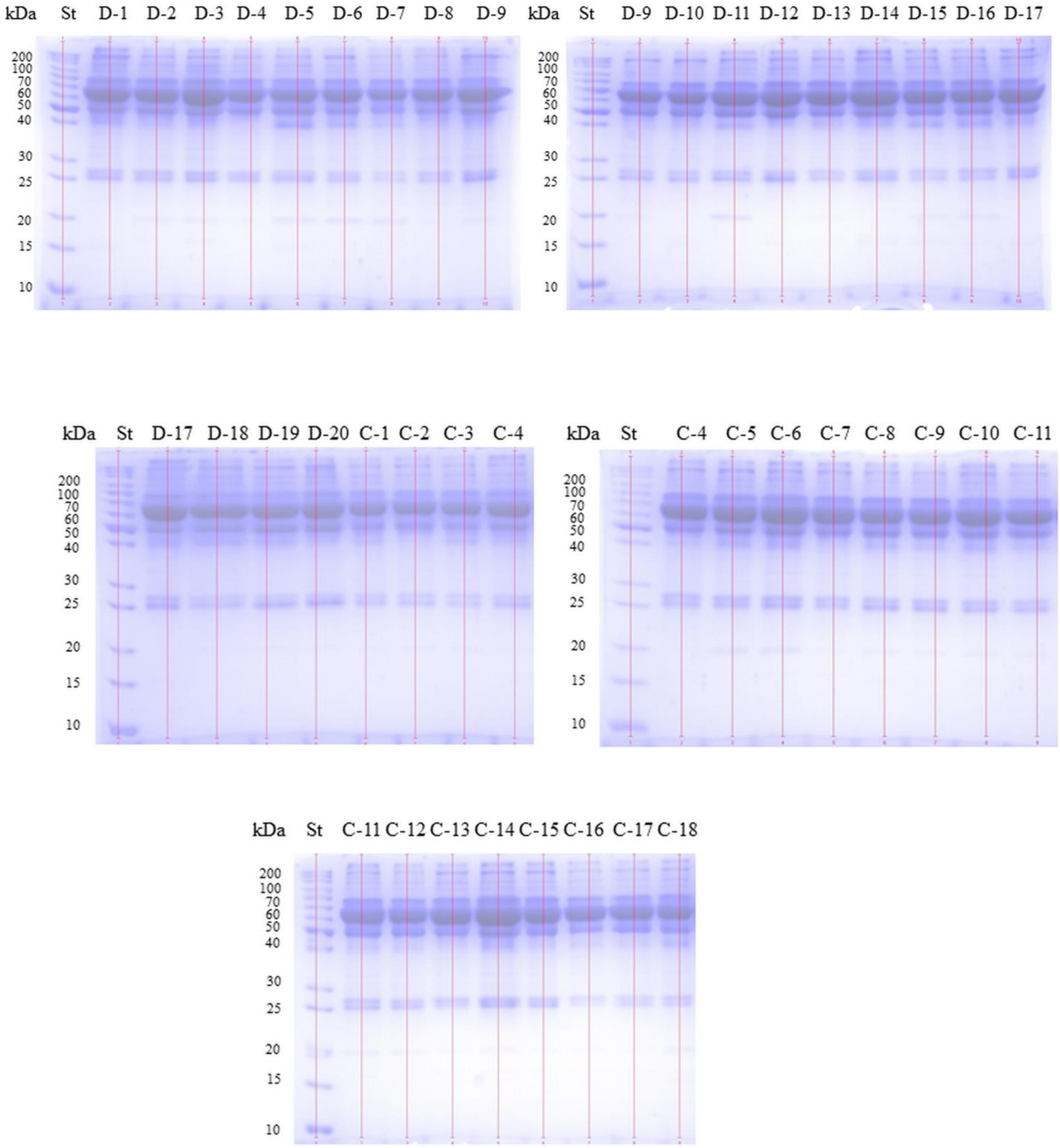

Figure S1 SDS-PAGE electropherograms of the individual plasma protein samples (load - 5 μg). D and C denote T2DM and control plasma samples, respectively, St denote protein ladder

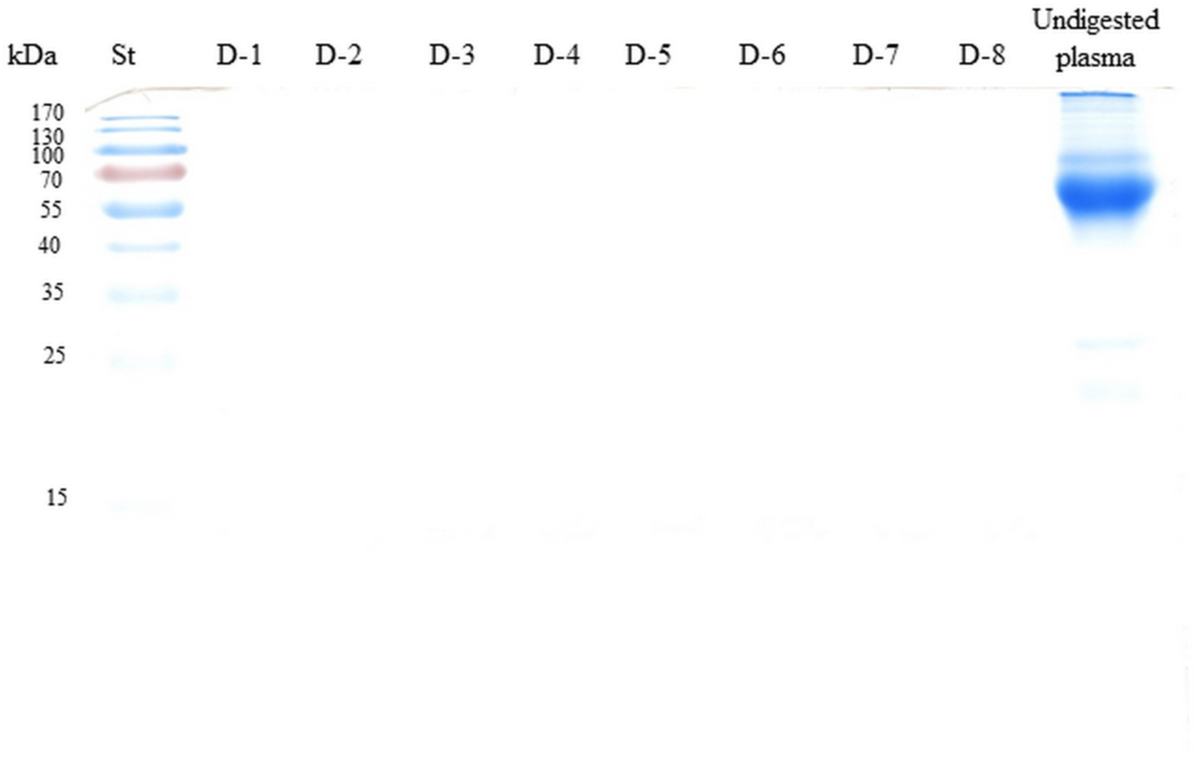

Figure S2 Example of a SDS-PAGE electrophoregram representing individual plasma protein tryptic digests (5 μg). St denote protein ladder

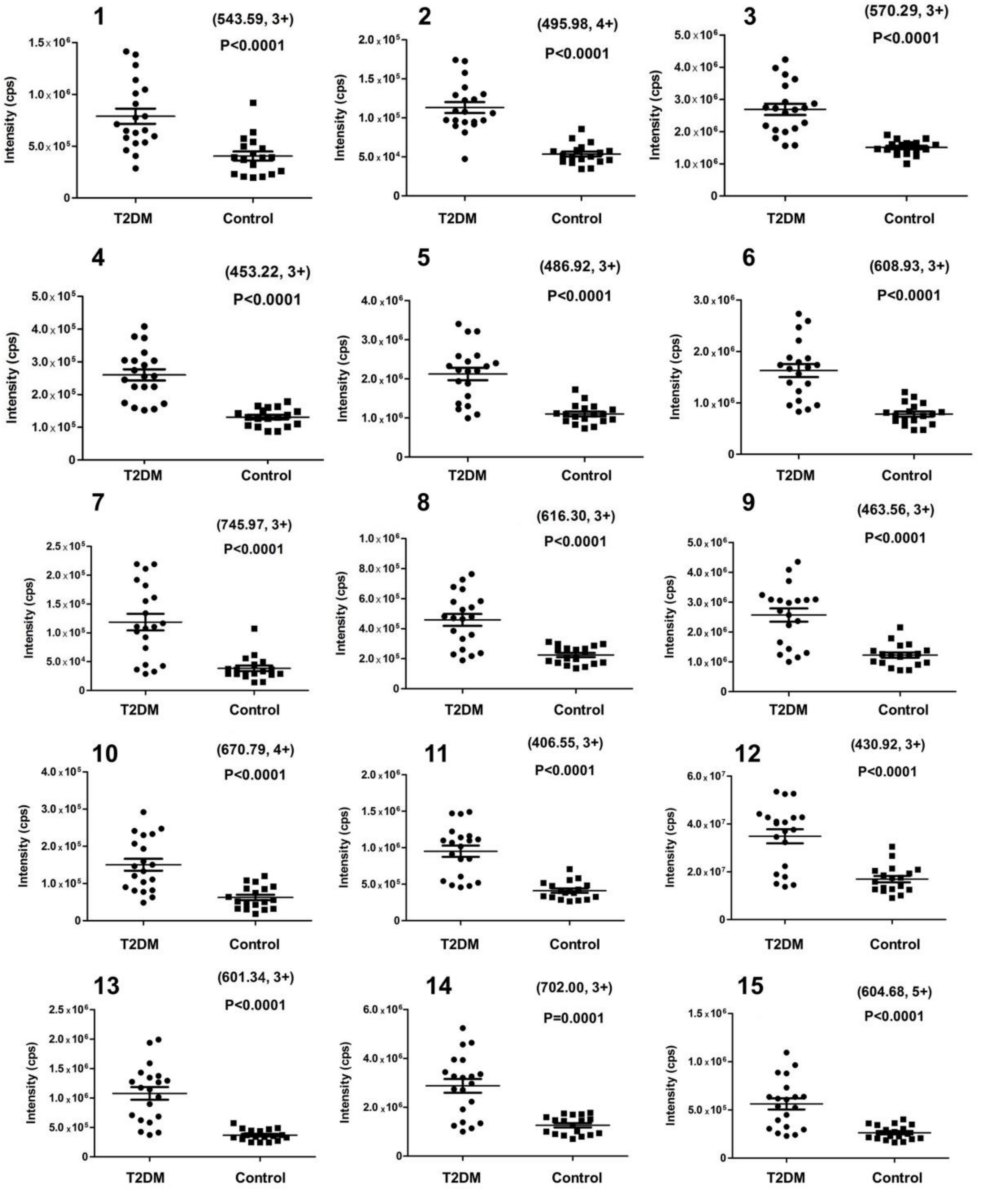

Figure S3 Integral peak areas observed for the peptides, demonstrating differential abundance in the T2DM and control tryptic digests. The data were acquired in the RP-UHPLC-QqTOF-MS experiments performed with the BAC-enriched tryptic digests obtained from individual T2DM and control plasma samples. Statistical

significance was estimated by the Mann-Whitney U-test, and the p values below 0.05 were considered to be significant

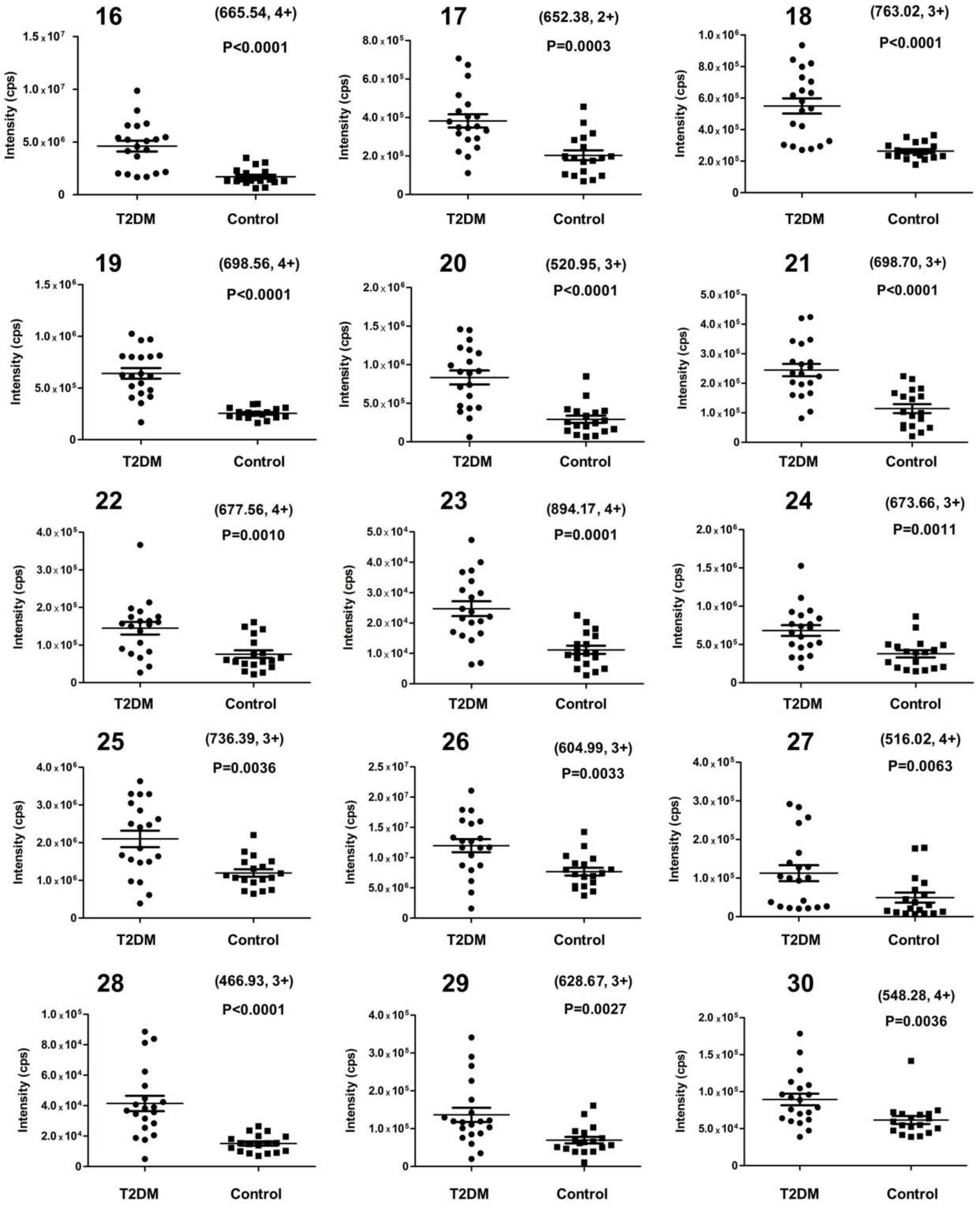

Figure S3 (Continued)

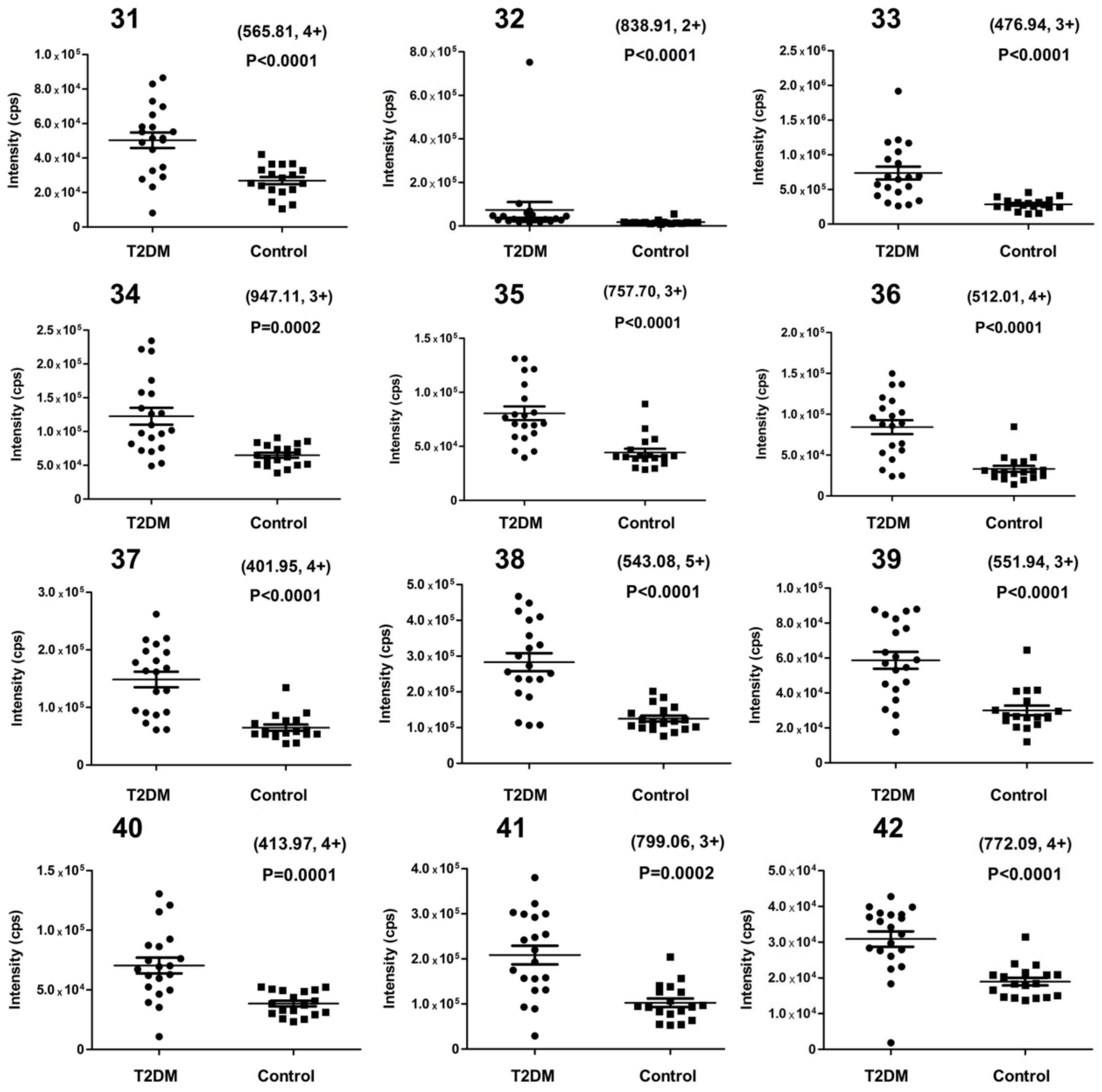

Figure S3 (Continued)

Figure S4 Tandem mass spectra of differentially glycated peptides identified by database search using Proteome Discoverer 1.4 software, Uniprot human database (downloaded 7.06.2016), and Mascot search engine. Sequences were confirmed by manual interpretation. K_{Am} and C_{CAM} denote glycated lysyl and carbamidomethylated cysteinyl residues, respectively.

respectively.

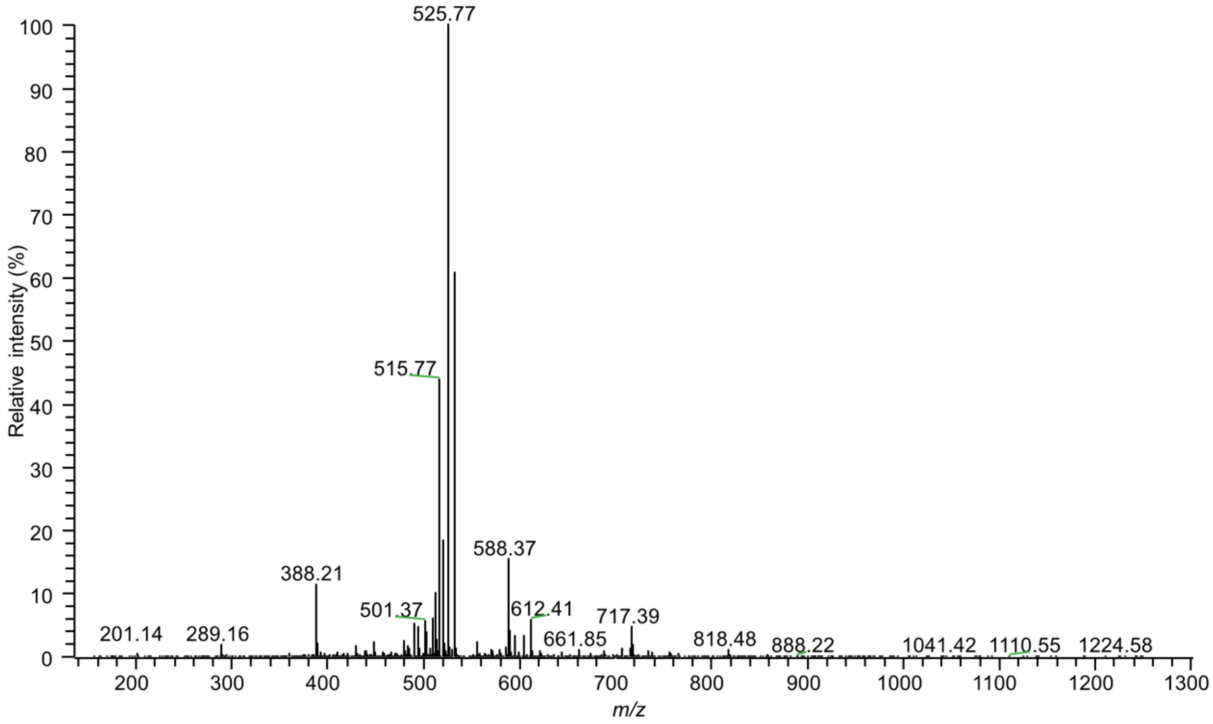

Figure S4-1 MS/MS spectrum of **VTK$_{Am}$C$_{CAM}$C$_{CAM}$TESLVNR**.

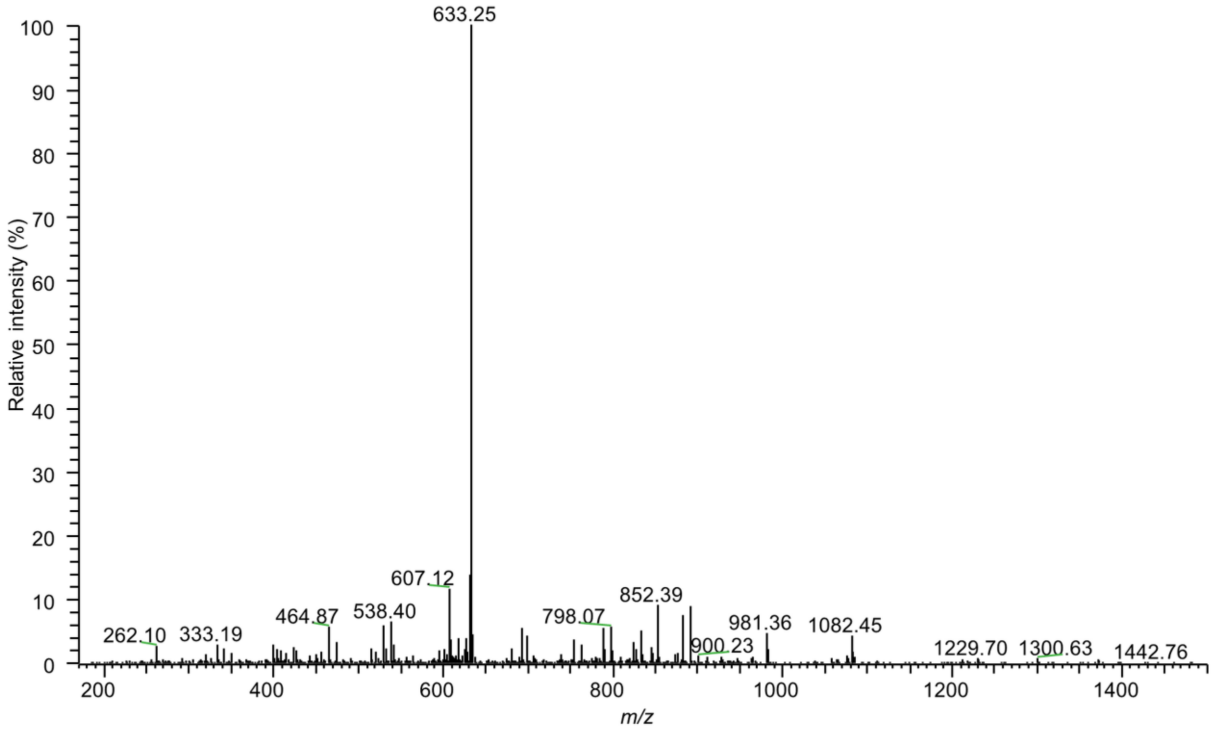

Figure S4-2 MS/MS spectrum of **RYK$_{\mathbf{Am}}$AAFTEC$_{\mathbf{CAM}}$C$_{\mathbf{CAM}}$QAADK**.

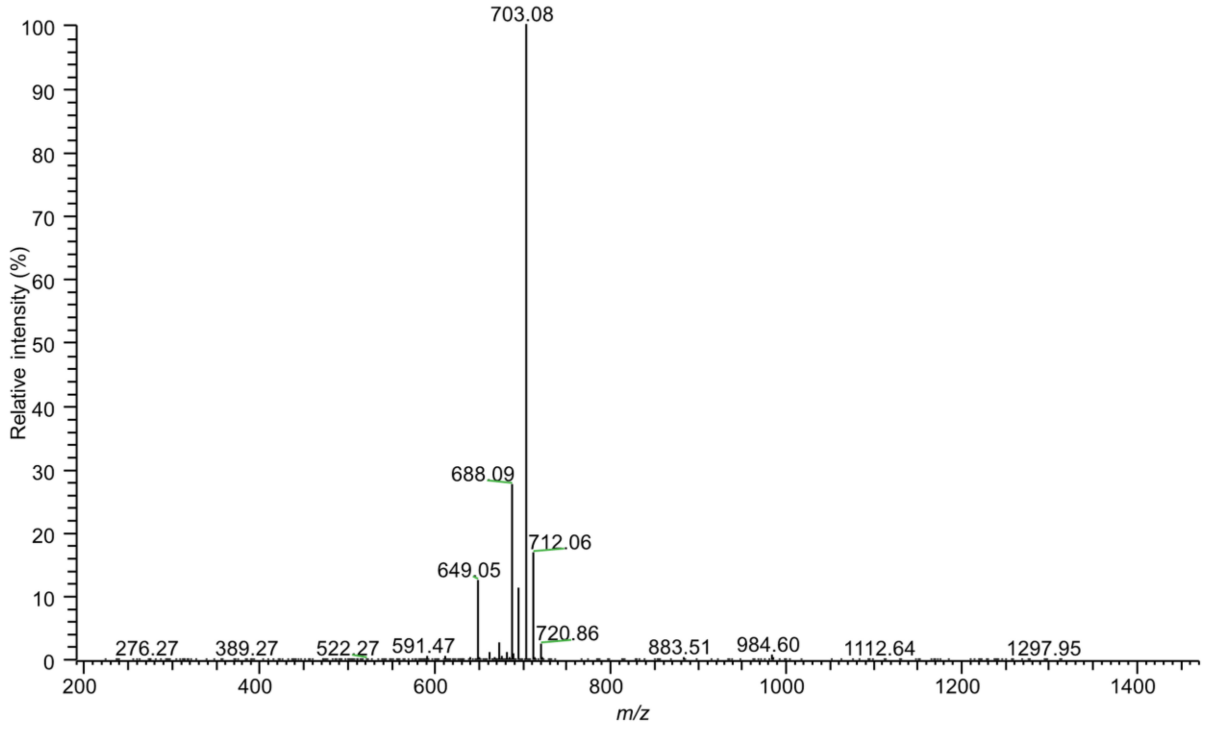

Figure S4-3 MS/MS spectrum of **LAK$_{\mathbf{Am}}$TYETTLEK**.

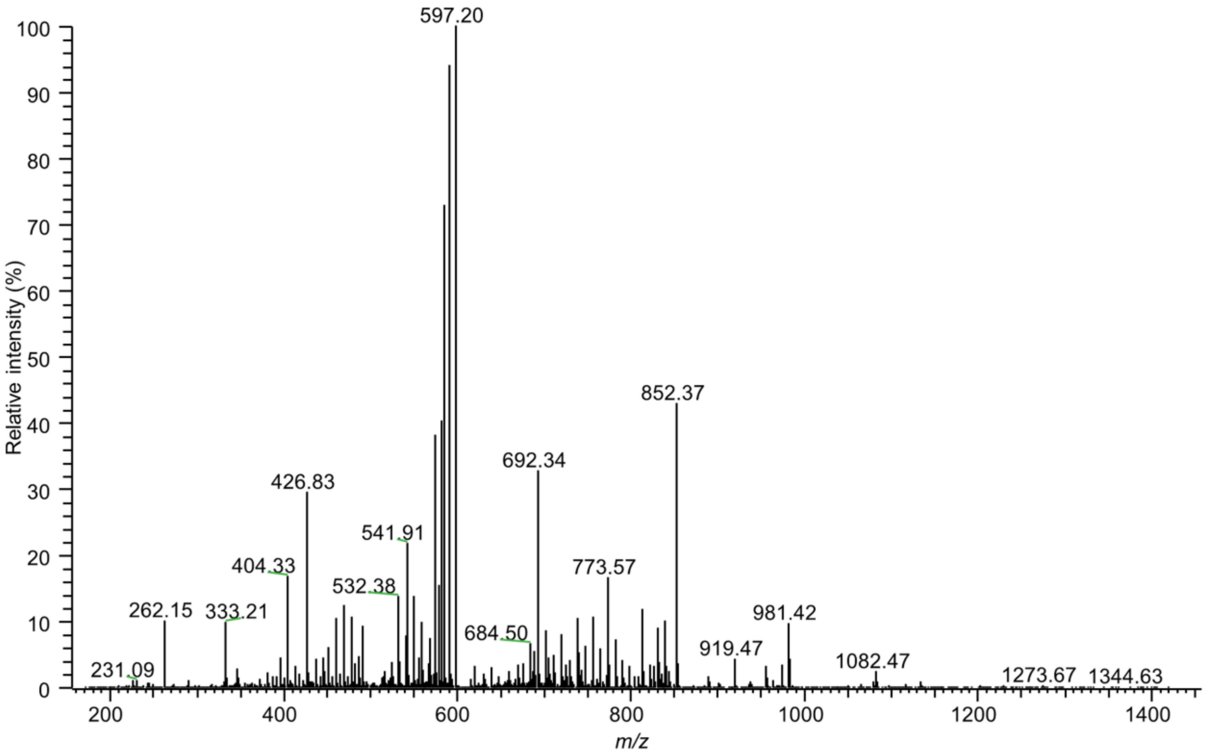

Figure S4-4 MS/MS spectrum of **YK$_{Am}$AAFTEC$_{CAM}$C$_{CAM}$QAADK**.

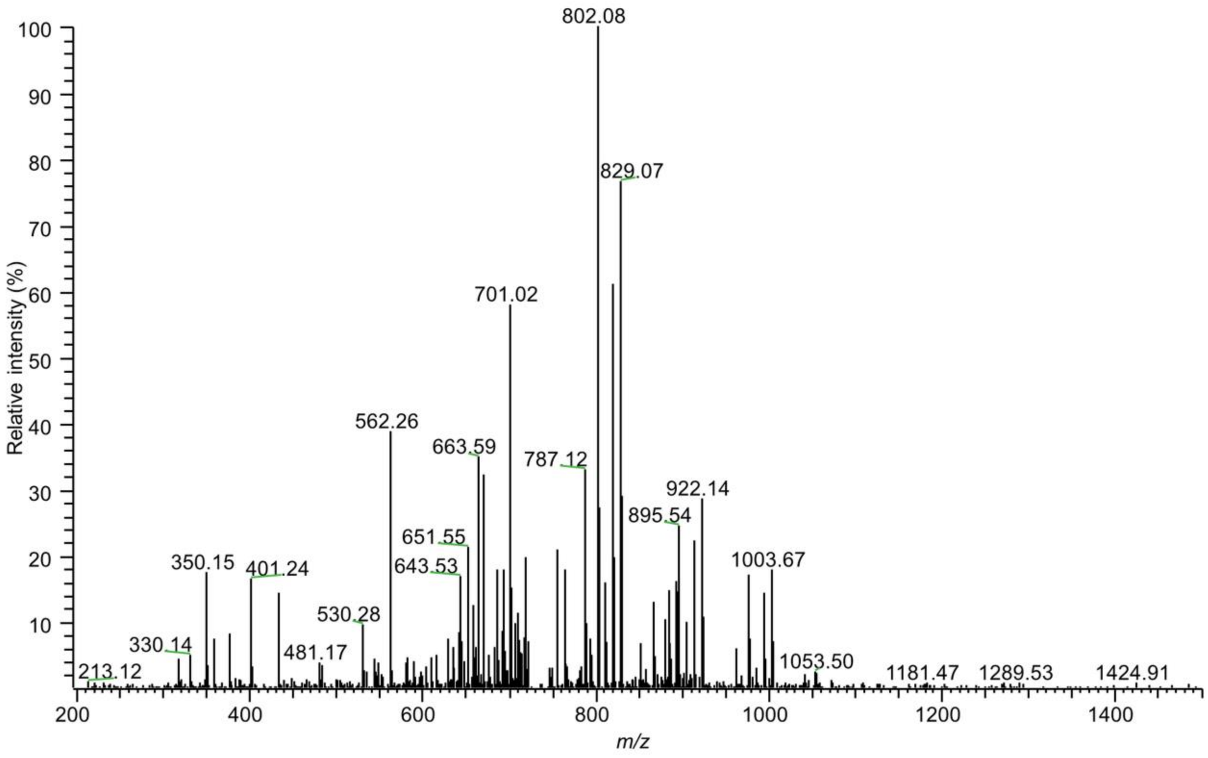

Figure S4-5 MS/MS spectrum of **ETYGEMADC$_{CAM}$C$_{CAM}$AK$_{Am}$QEPER**.

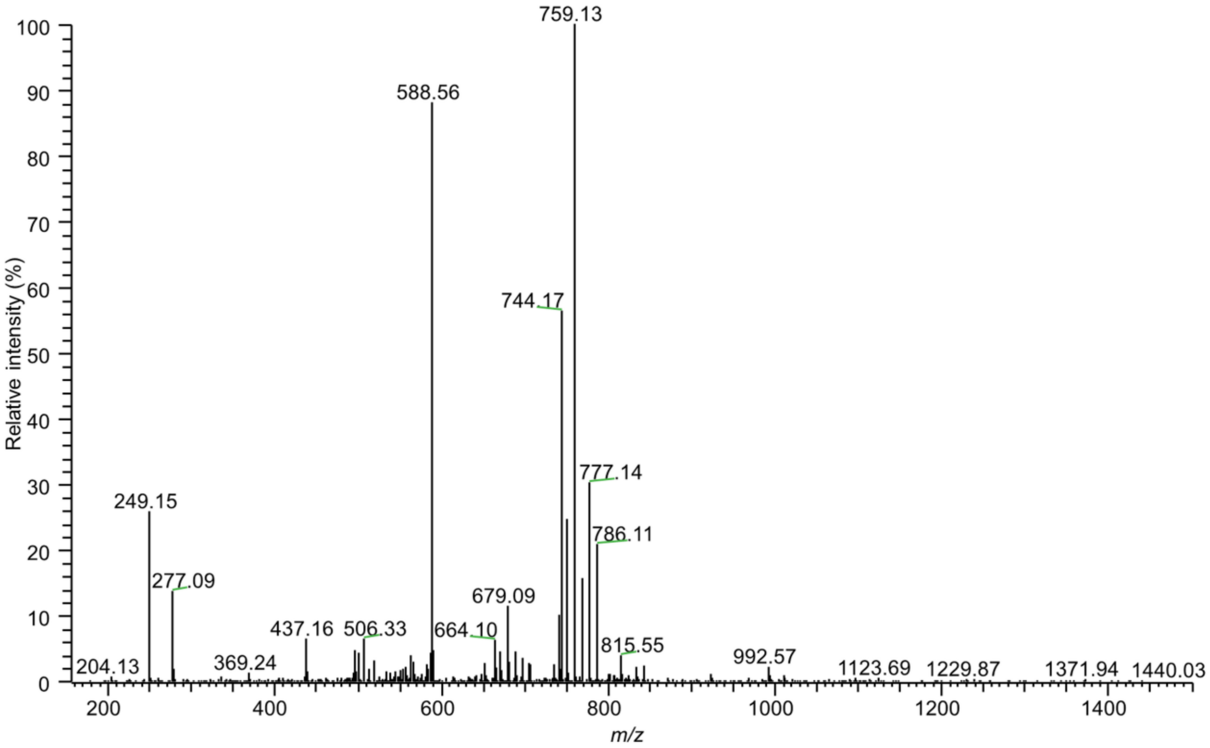

Figure S4-6 MS/MS spectrum of **YIC$_{CAM}$ENQDSISSK$_{Am}$LK**.

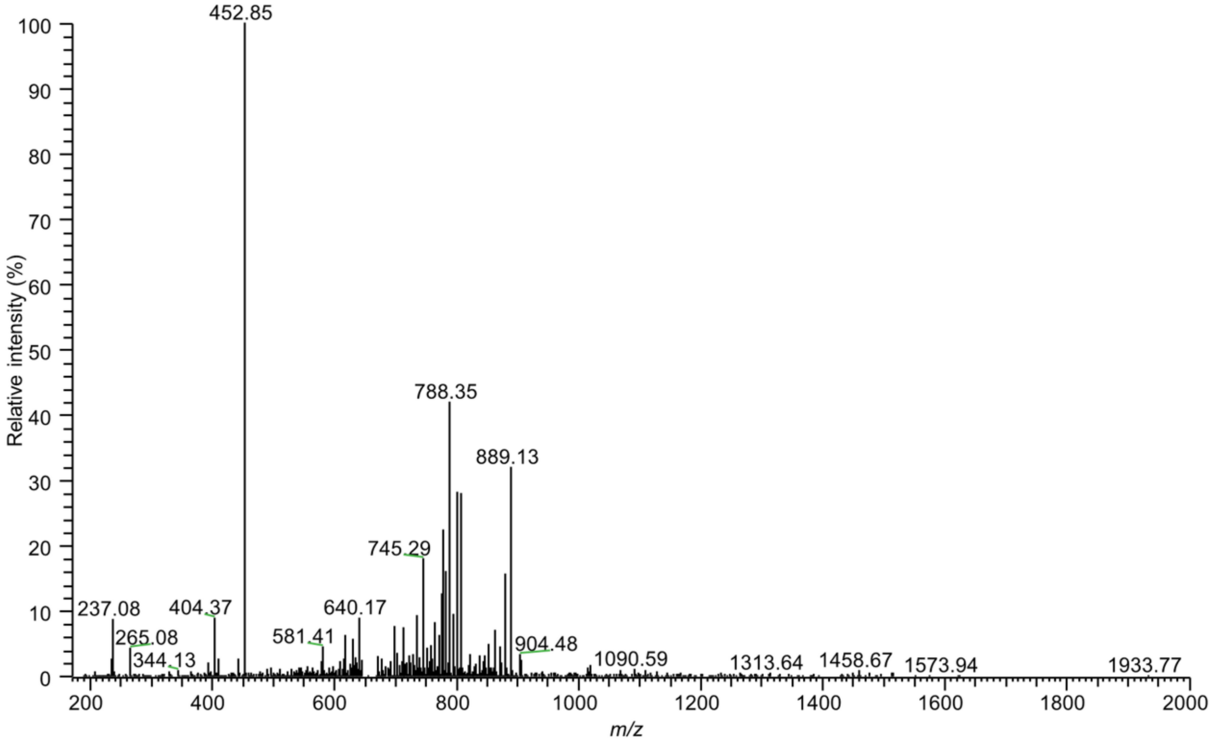

Figure S4-7 MS/MS spectrum of **TYETTLEK_{Am}C_{CAM}C_{CAM}AAADPHEC_{CAM}YAK**

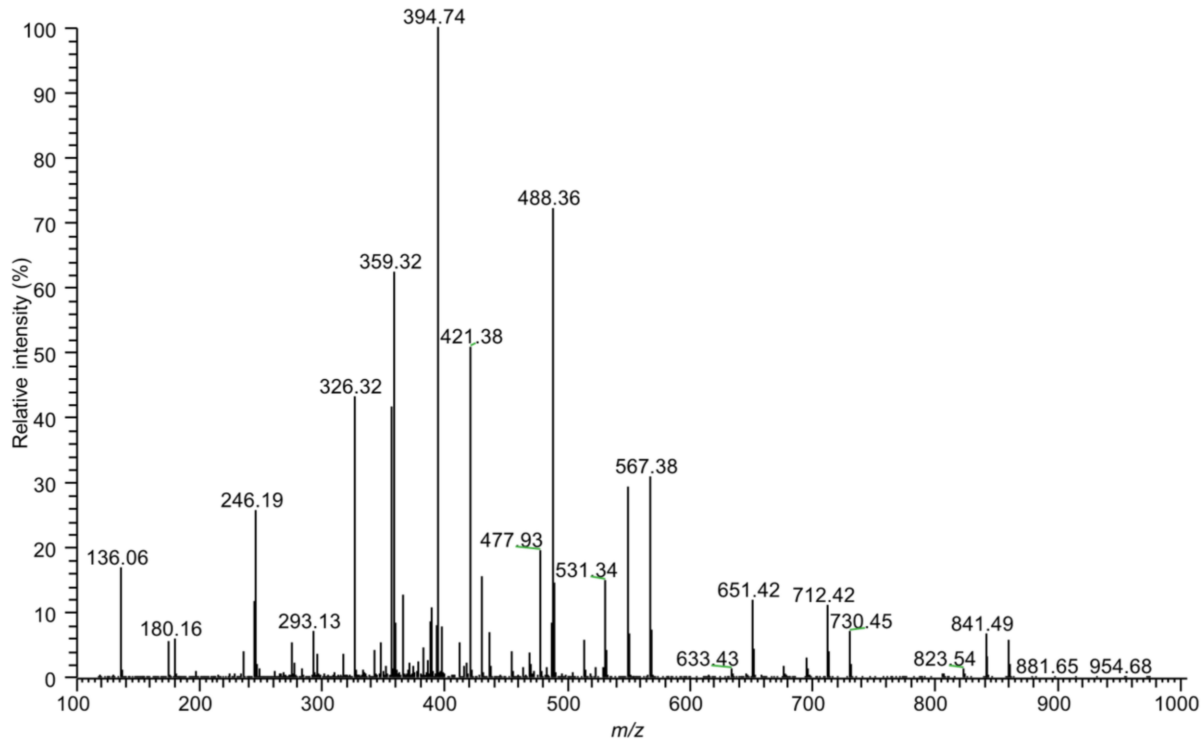

Figure S4-8 MS/MS spectrum of **K_{Am}YLYEIAR**

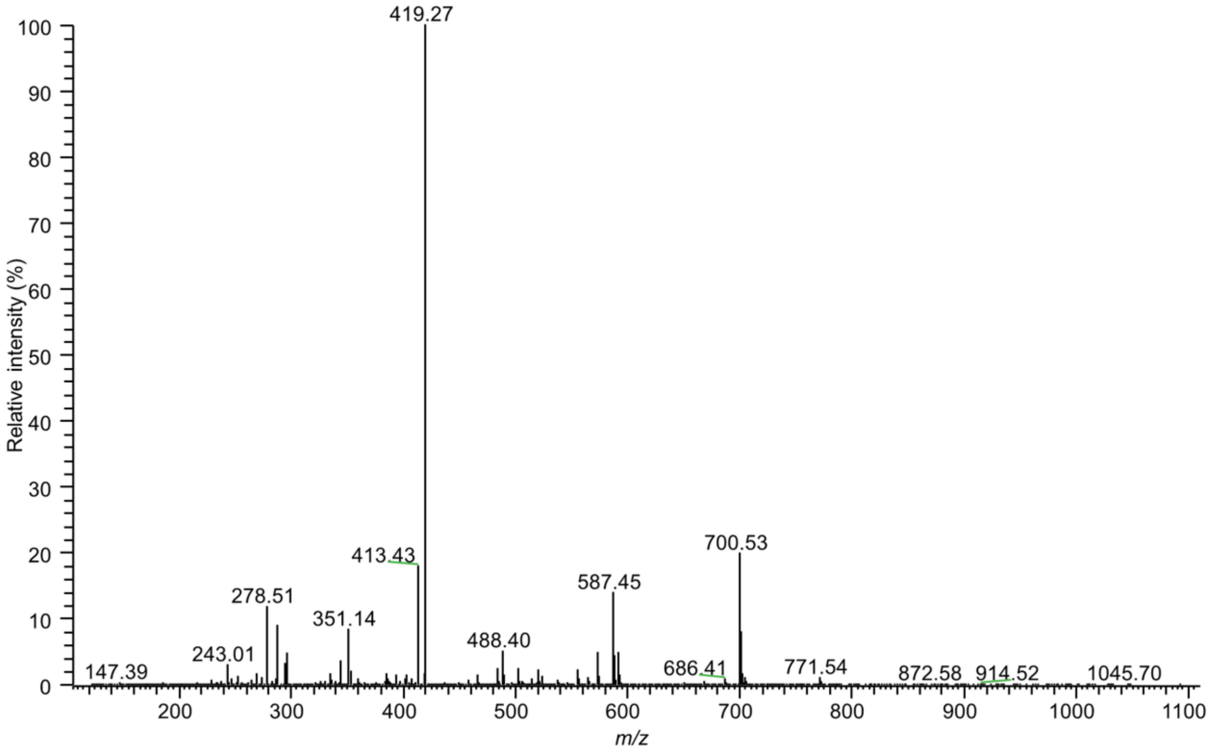

Figure S4-9 MS/MS spectrum of **K$_{\mathrm{Am}}$QTALVELVK**

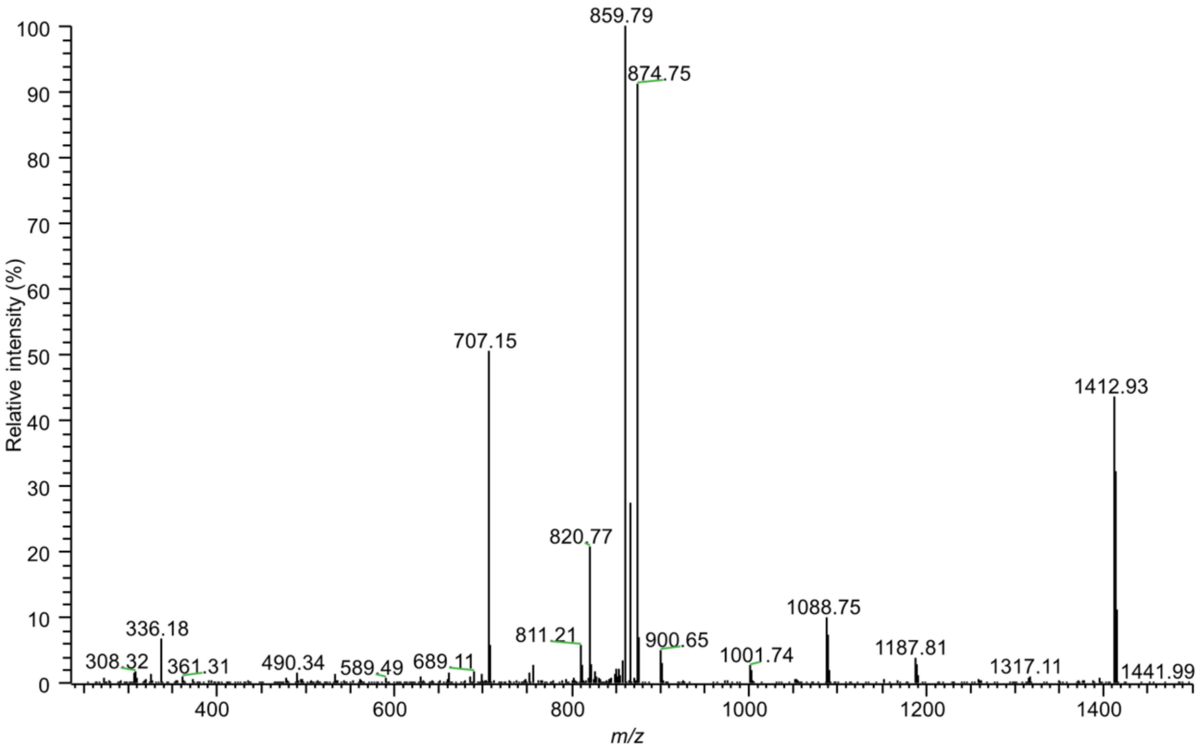

Figure S4-10 MS/MS spectrum of **K$_{\mathrm{Am}}$VPQVSTPTLVEVSR**

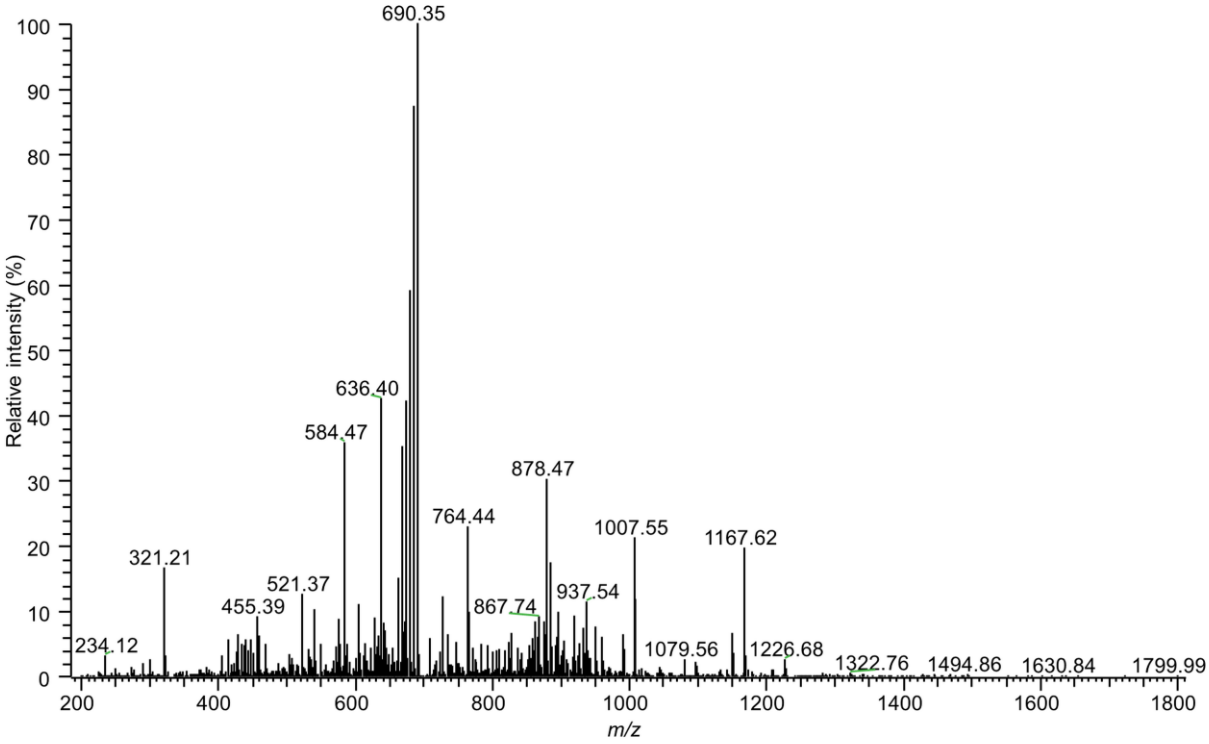

Figure S4-11 MS/MS spectrum of **ADLAK$_{\mathrm{Am}}$YIC$_{\mathrm{CAM}}$ENQDSISSK**

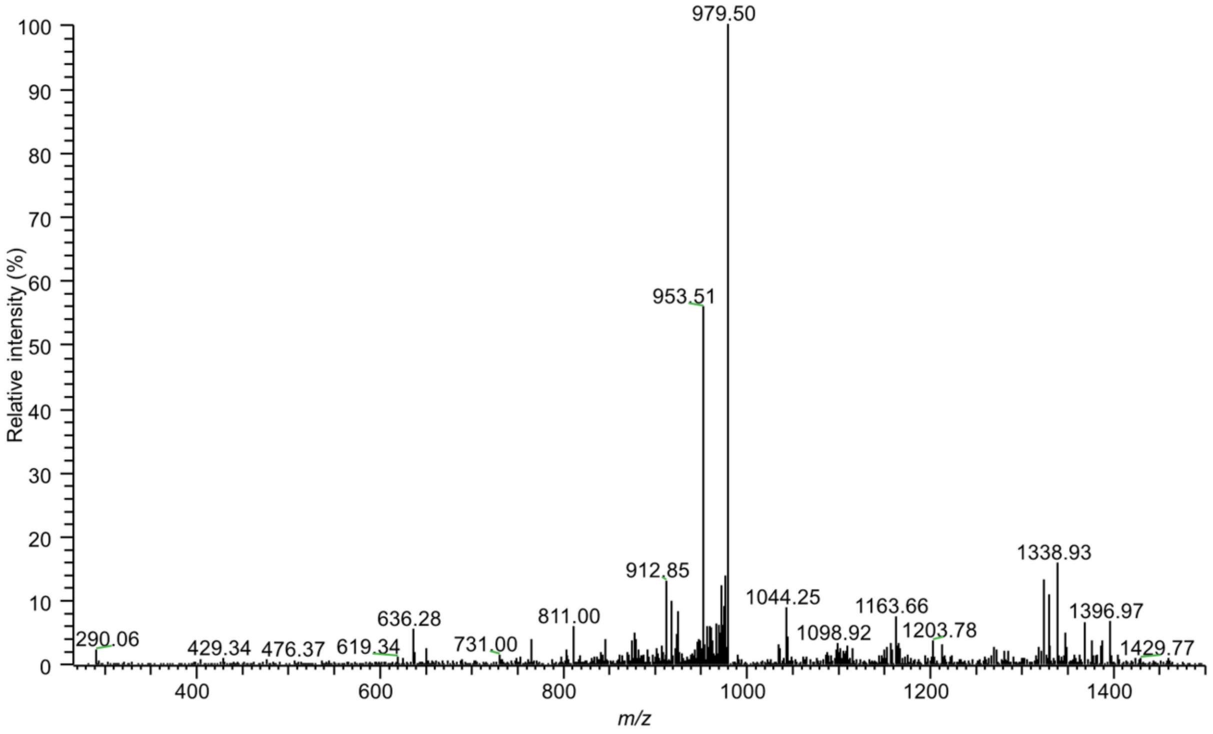

Figure S4-12 MS/MS spectrum of **LVTDLTK**$_{\mathrm{Am}}$**VHTEC**$_{\mathrm{CAM}}$**C**$_{\mathrm{CAM}}$**HGDLLEC**$_{\mathrm{CAM}}$**ADDR**

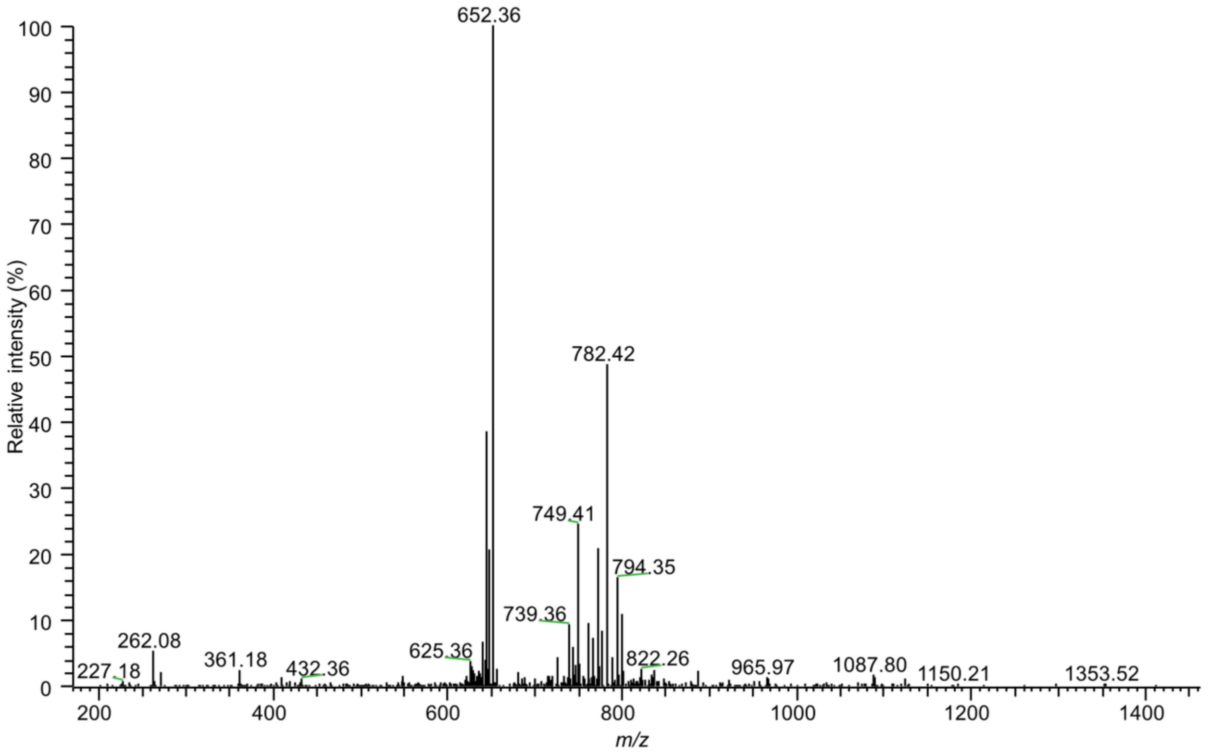

Figure S4-13 MS/MS spectrum of **TC**$_{\mathrm{CAM}}$**VADESAENC**$_{\mathrm{CAM}}$**DK**$_{\mathrm{Am}}$**SLHTLFGDK**

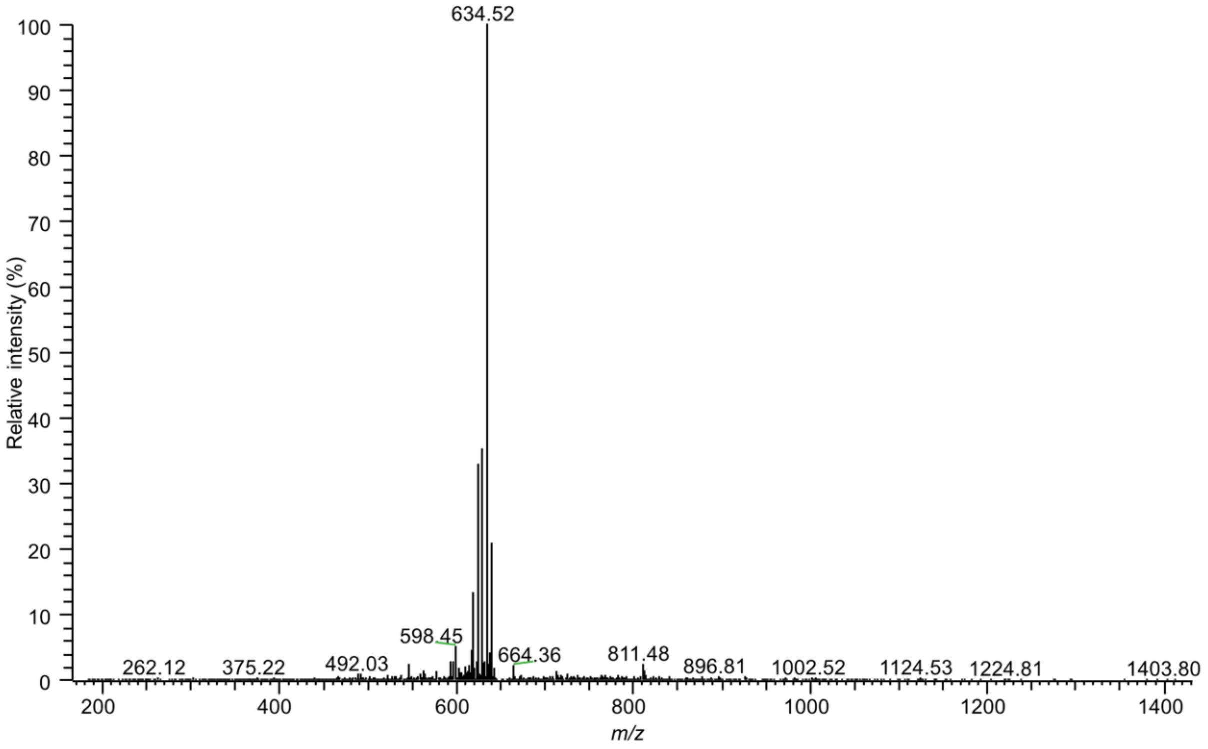

Figure S4-14 MS/MS spectrum of **K$_{\mathbf{Am}}$LVAASQAALGL**

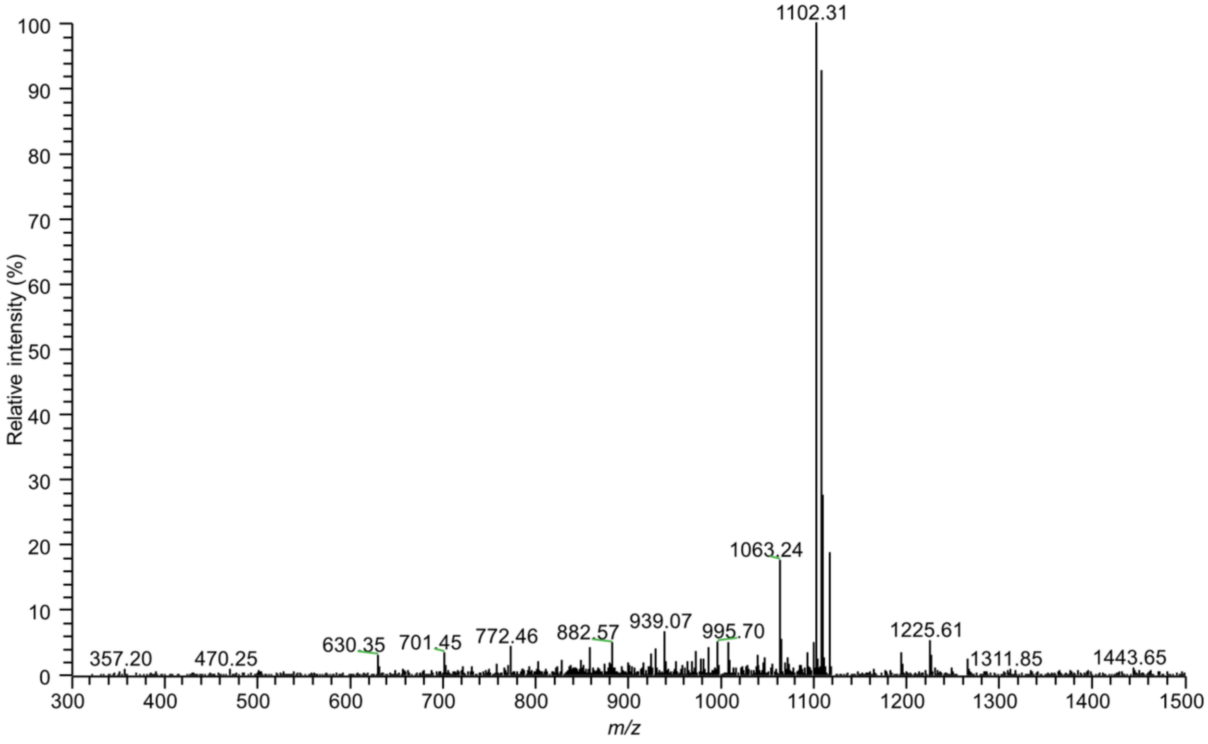

Figure S4-15 MS/MS spectrum of **AAFTEC$_{\mathbf{CAM}}$C$_{\mathbf{CAM}}$QAADK$_{\mathbf{Am}}$AAC$_{\mathbf{CAM}}$LLPK**

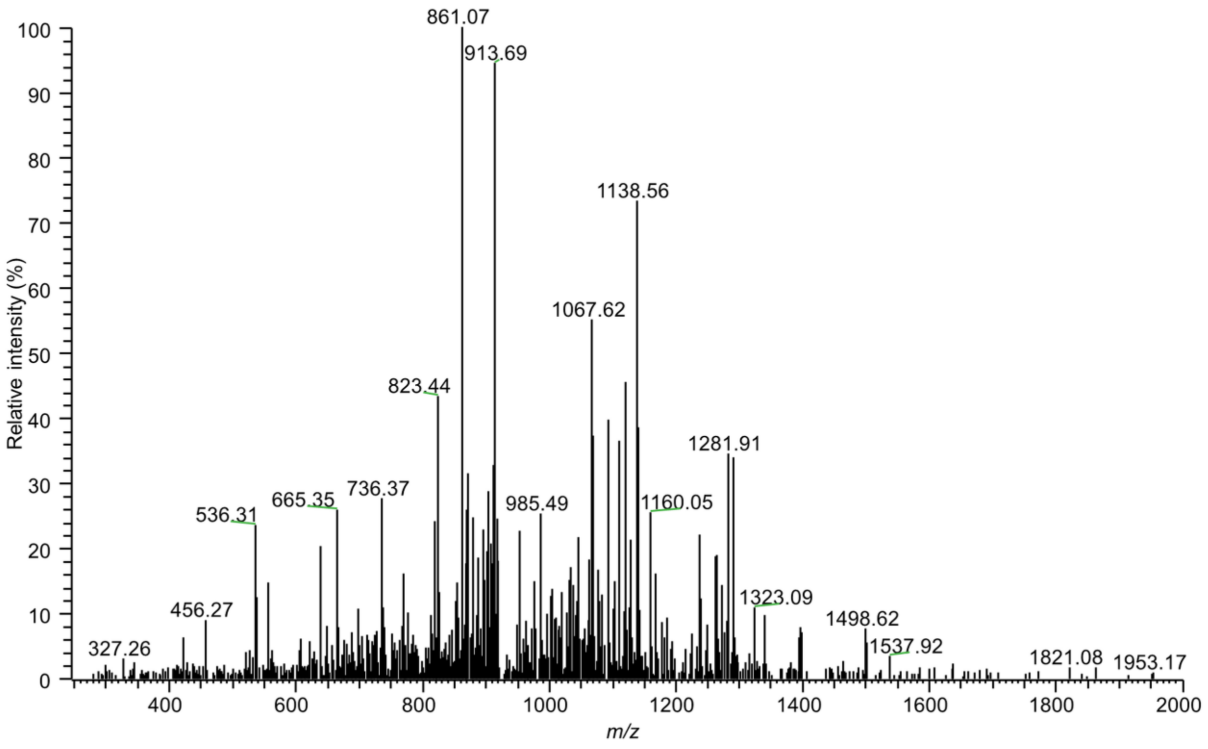

Figure S4-16 MS/MS spectrum of **LVNEVTEFAK$_{Am}$TC$_{CAM}$VADESAENC$_{CAM}$DK**

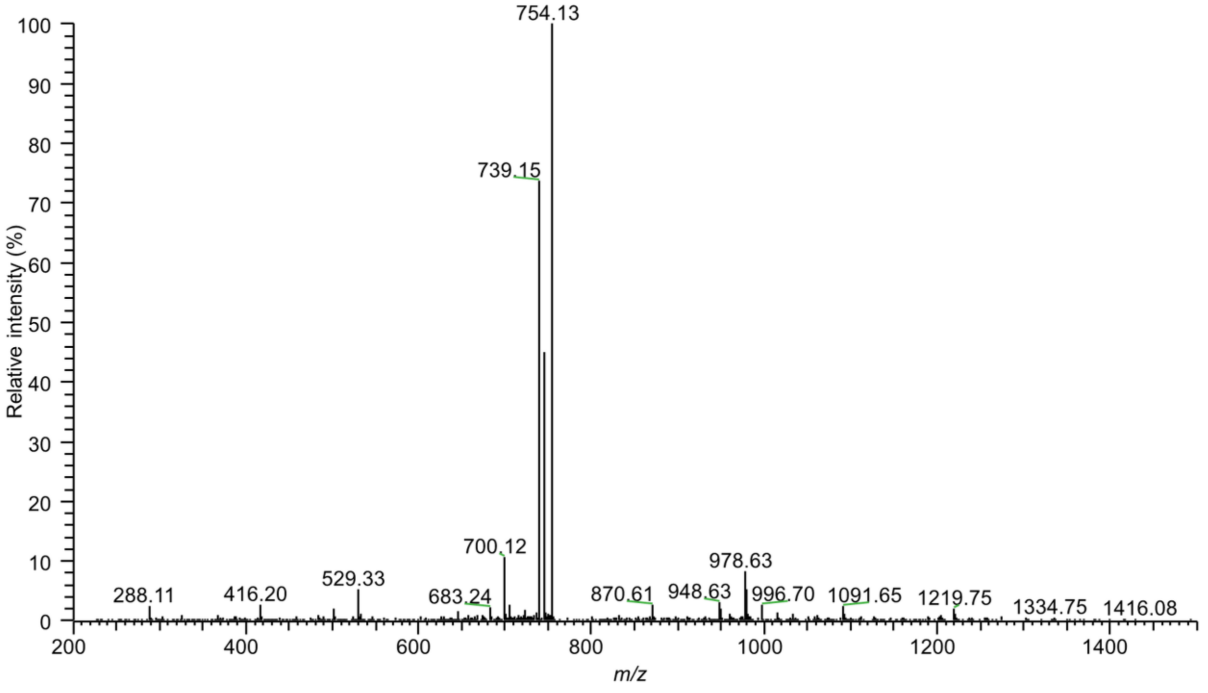

Figure S4-17 MS/MS spectrum of **AAC$_{CAM}$LLPK$_{Am}$LDELR**

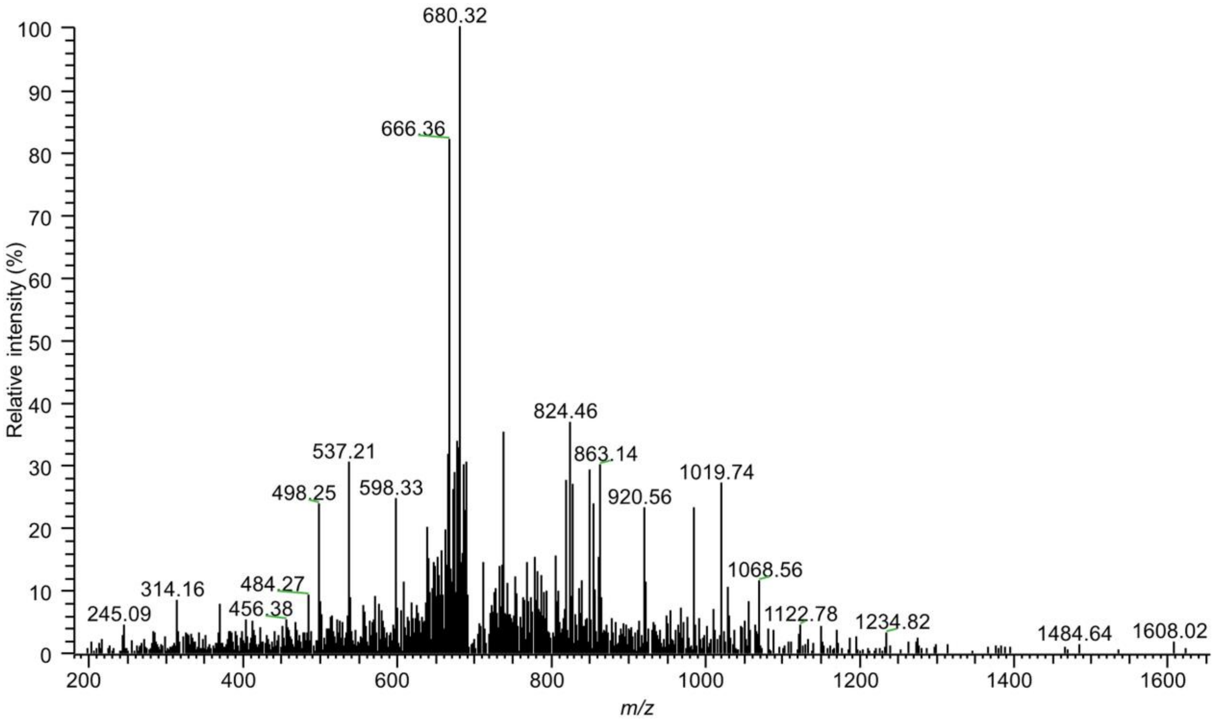

Figure S4-18 MS/MS spectrum of **SLHTLFGDK$_{Am}$LC$_{CAM}$TVATLR**

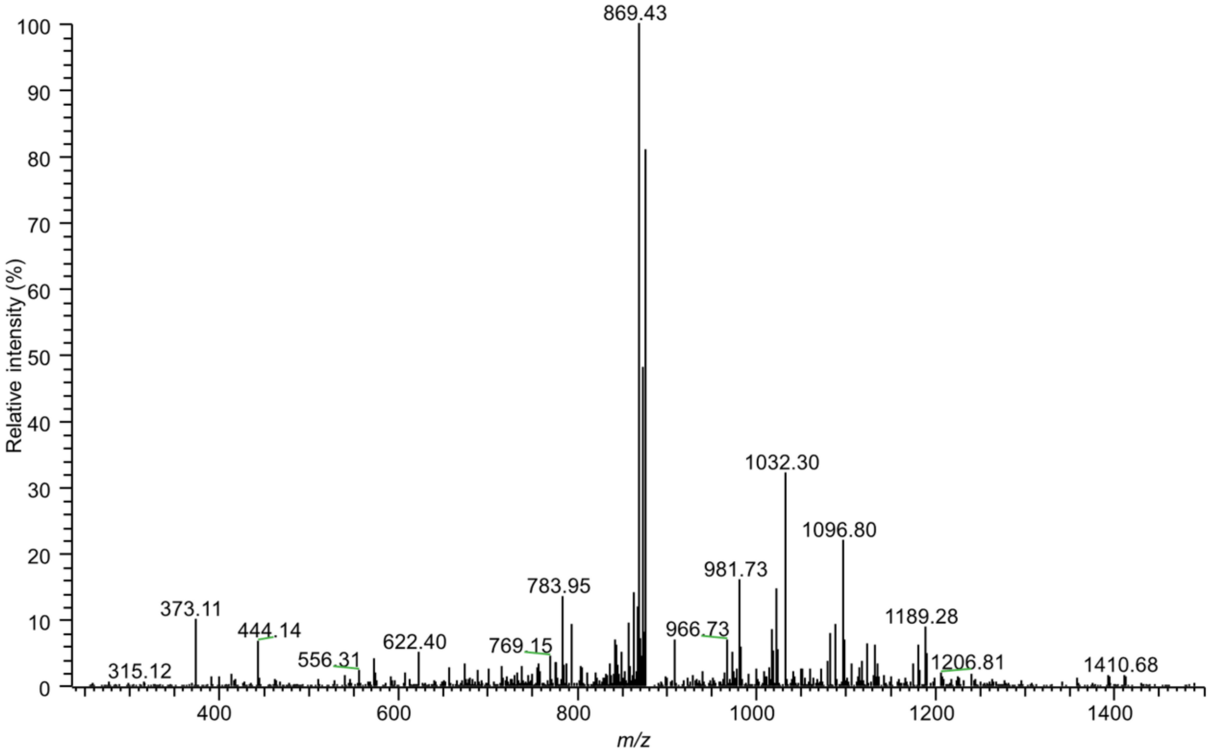

Figure S4-19 MS/MS spectrum of **EFNAETFTFHADIC$_{CAM}$TLSEK$_{Am}$ER**

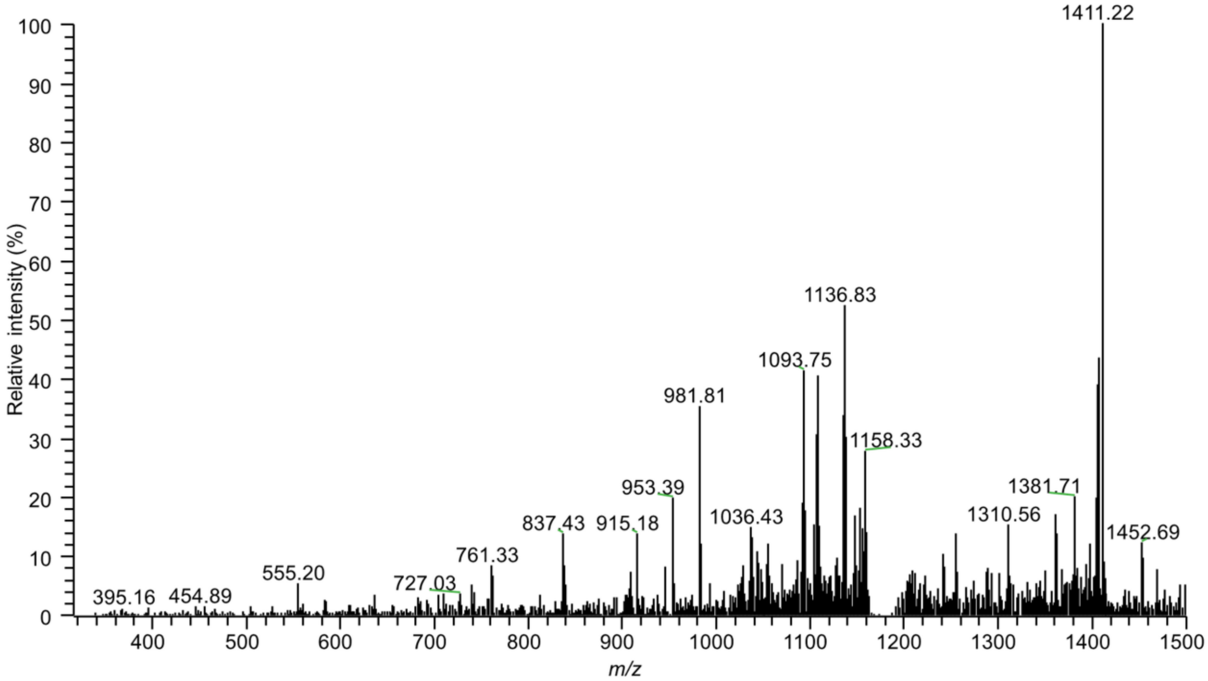

Figure S4-20 MS/MS spectrum of

TC$_{\mathrm{CAM}}$VADESAENC$_{\mathrm{CAM}}$DK$_{\mathrm{Am}}$SLHTLFGDKLC$_{\mathrm{CAM}}$TVATLR

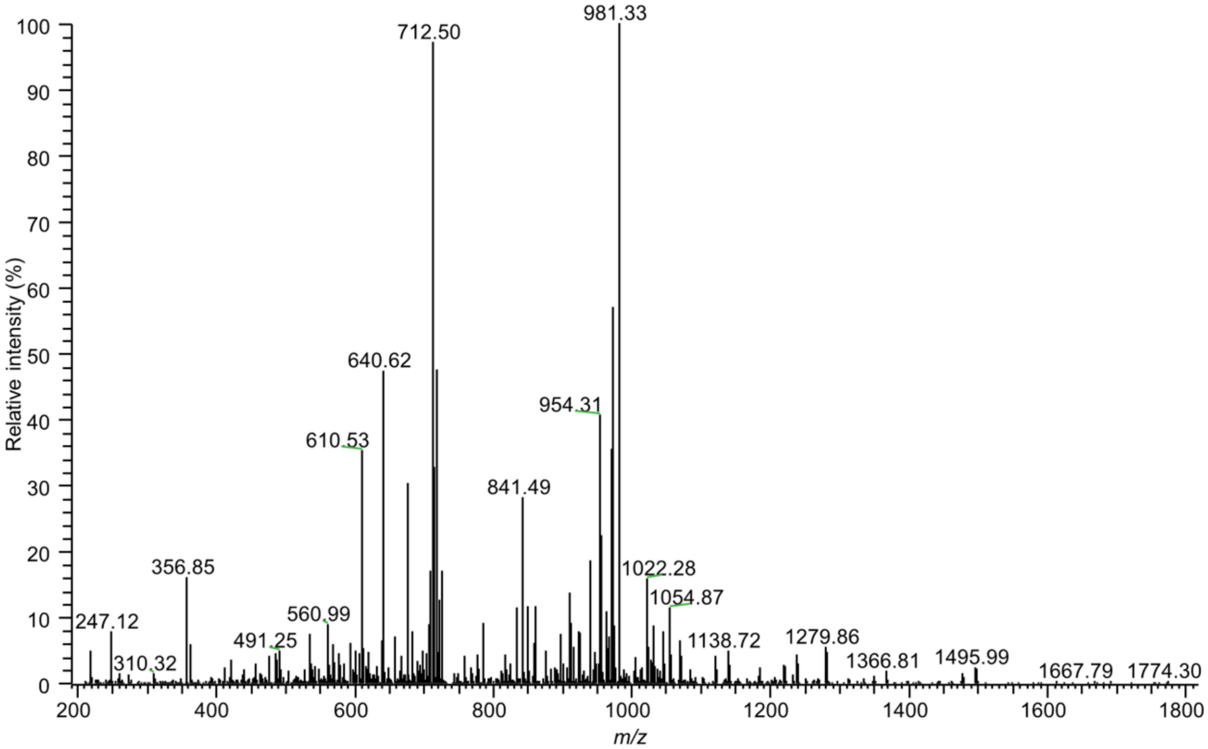

Figure S4-21 MS/MS spectrum of **VFDEFK$_{\mathrm{Am}}$PLVEEPQNLIK**

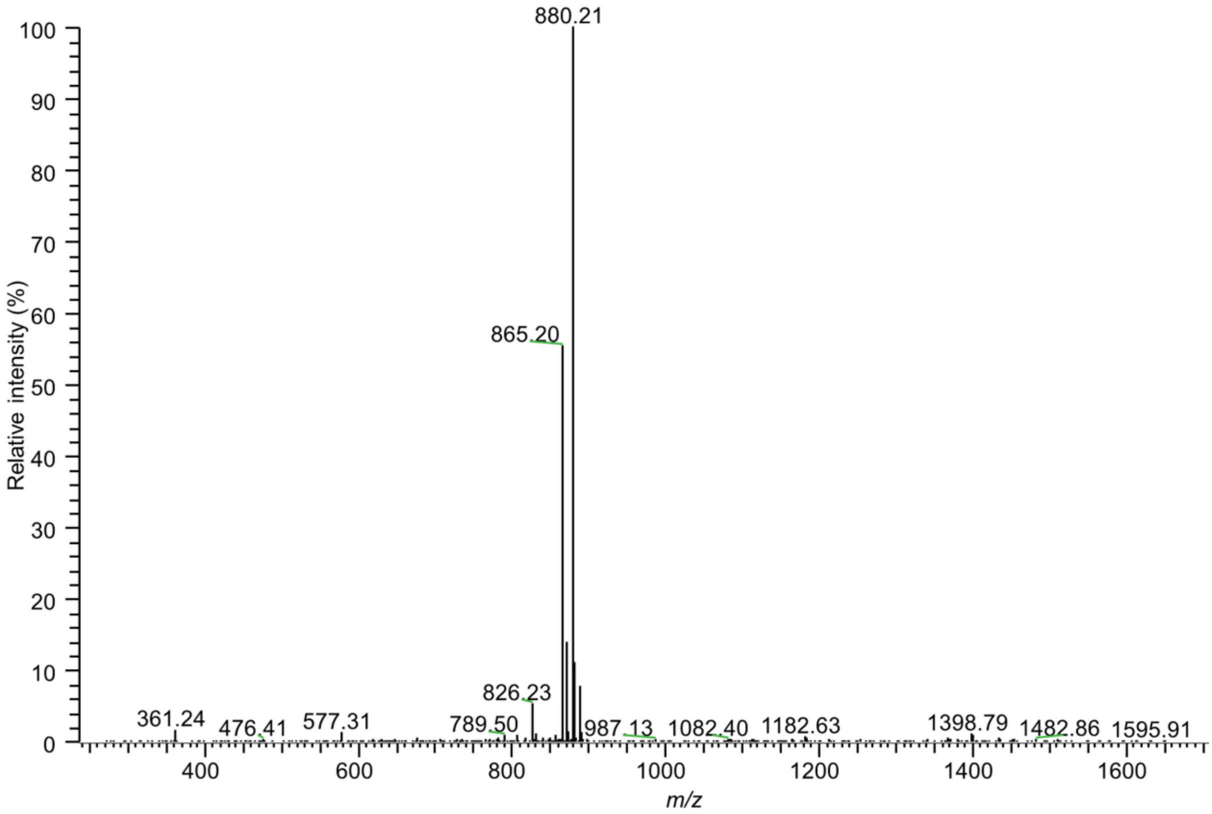

Figure S4-22 MS/MS spectrum of **AEFAEVSK**$_{\mathbf{Am}}$**LVTDLTK**

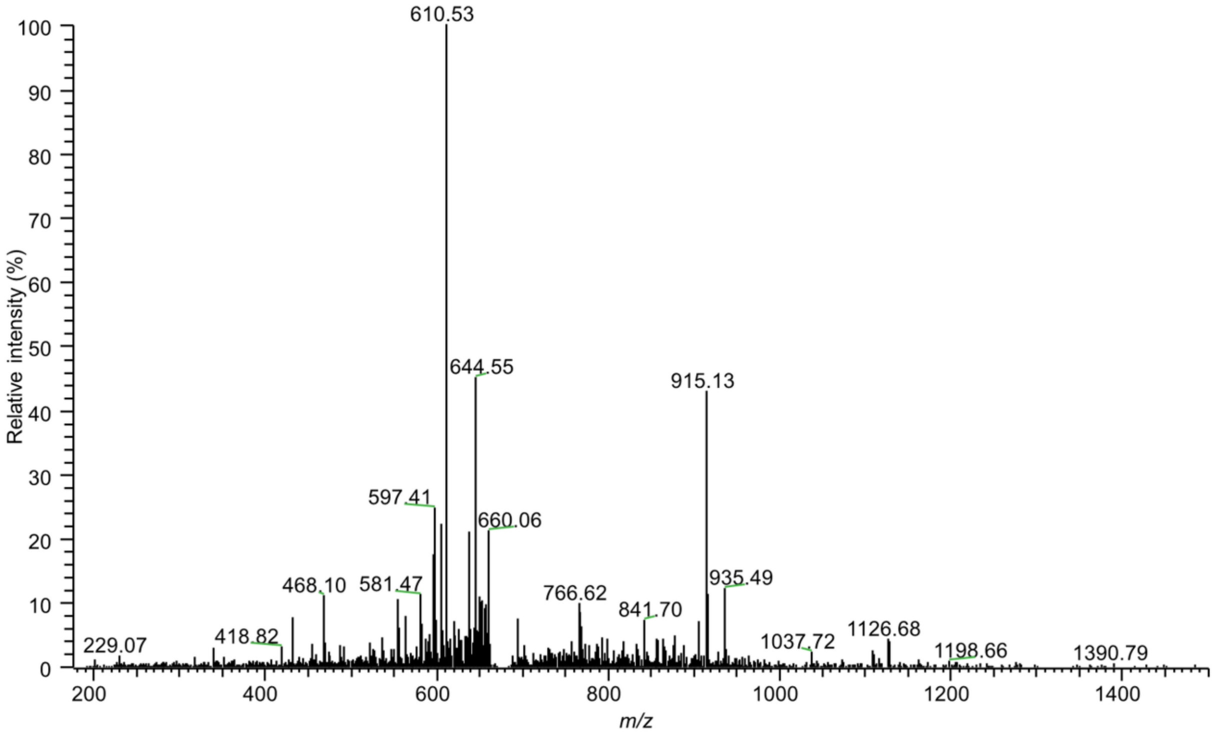

Figure S4-23 MS/MS spectrum of **RHPYFYAPELLFFAK**$_{\mathbf{Am}}$

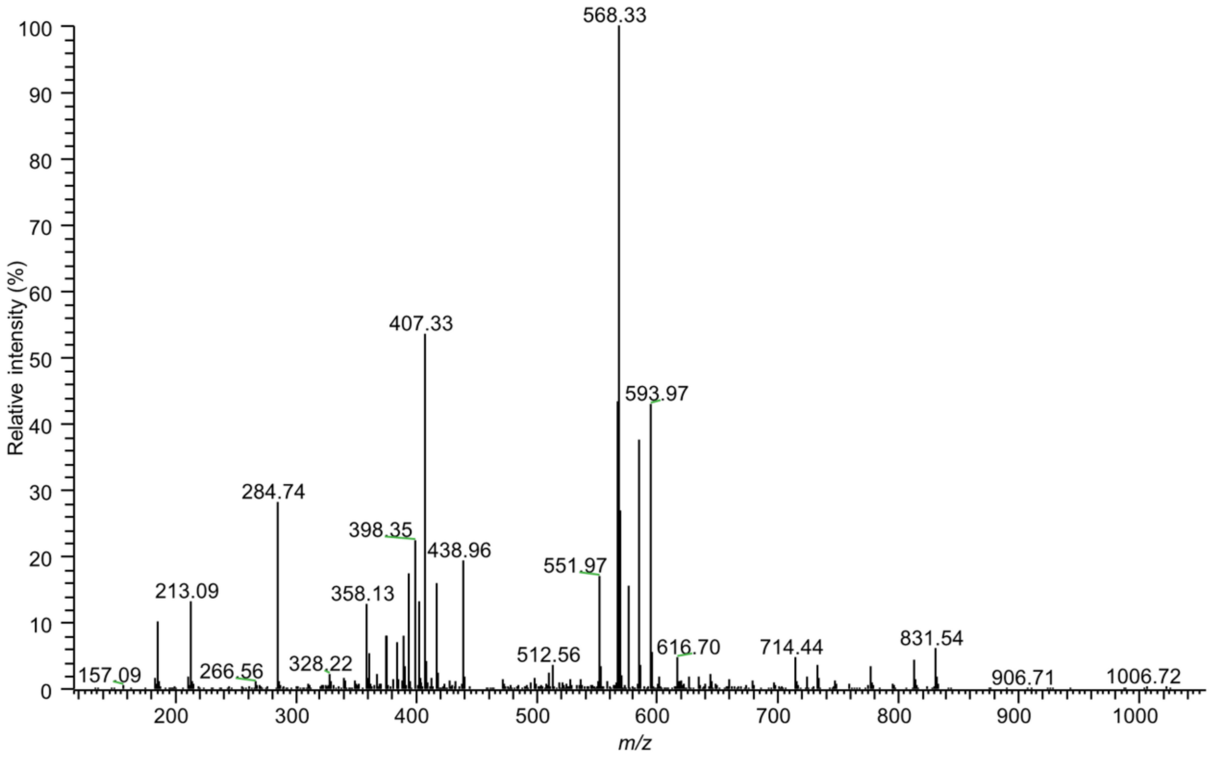

Figure S4-24 MS/MS spectrum of **LVDGK**$_{\mathbf{Am}}$**GVPIPNK**

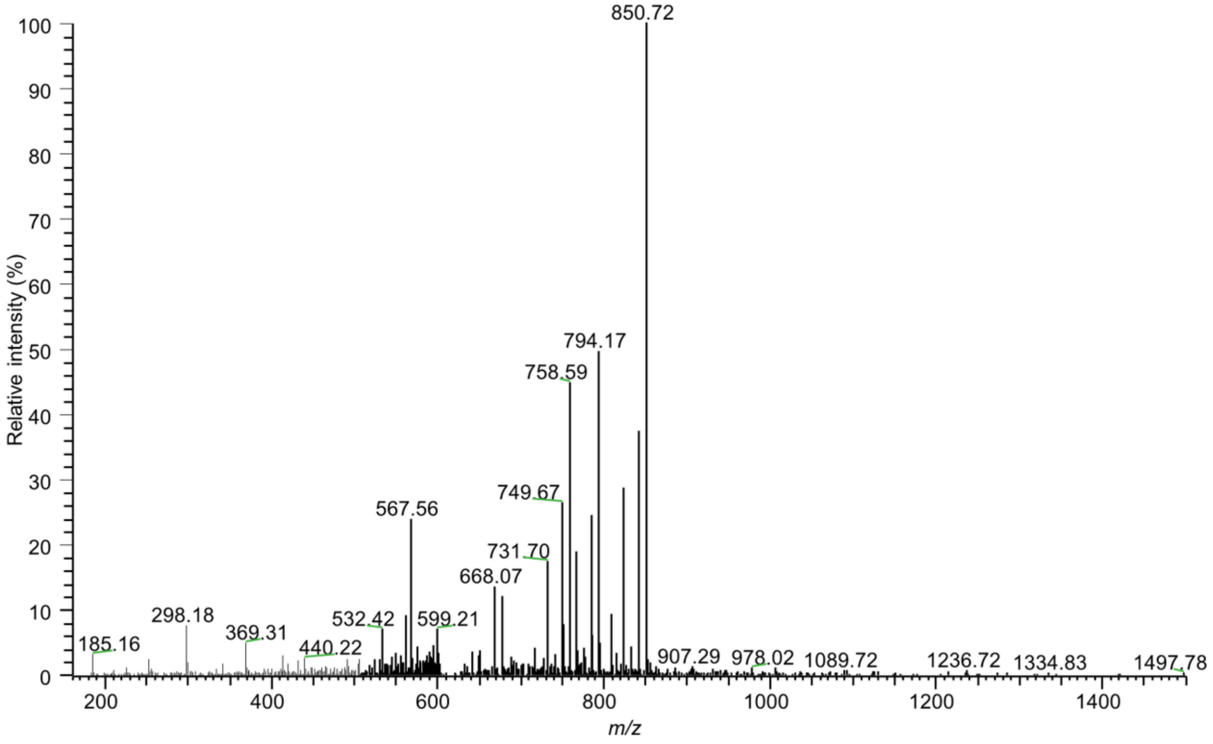

Figure S4-25 MS/MS spectrum of **ALLAYAFALAGNQDK**$_{\mathbf{Am}}$**R**

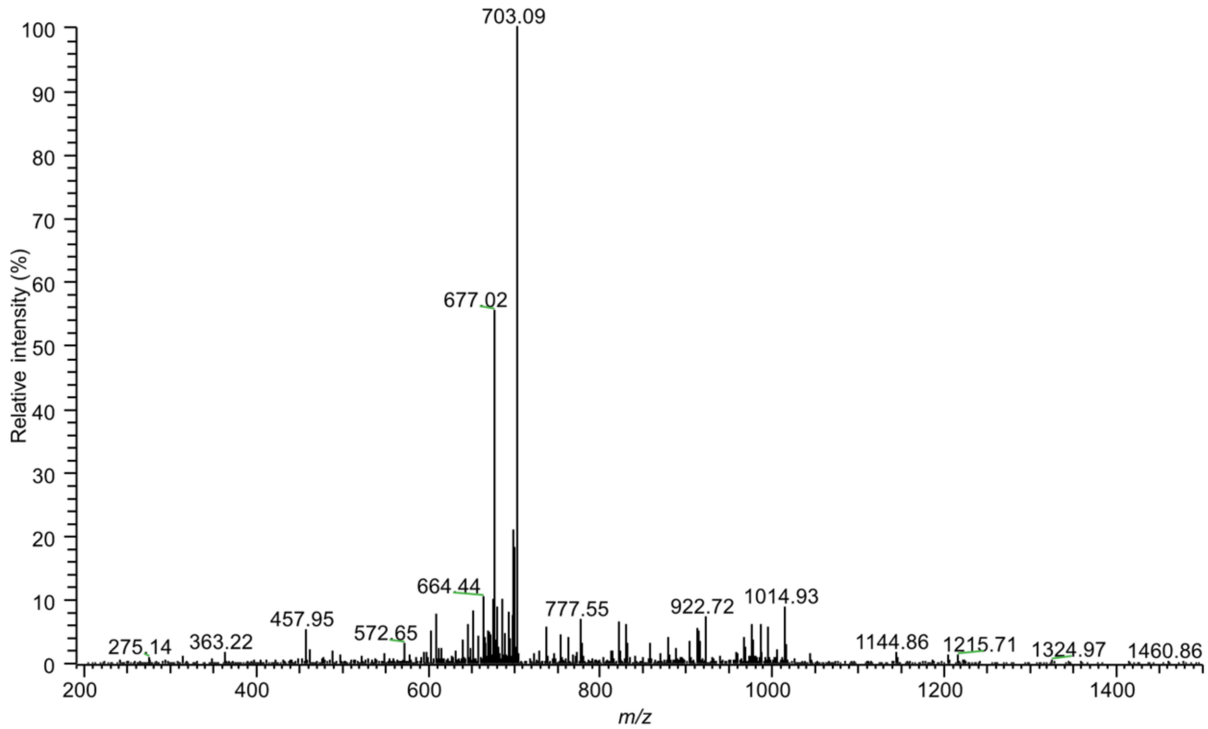

Figure S4-26 MS/MS spectrum of **LAEYHAK$_{\mathbf{Am}}$ATEHLSTLSEK**

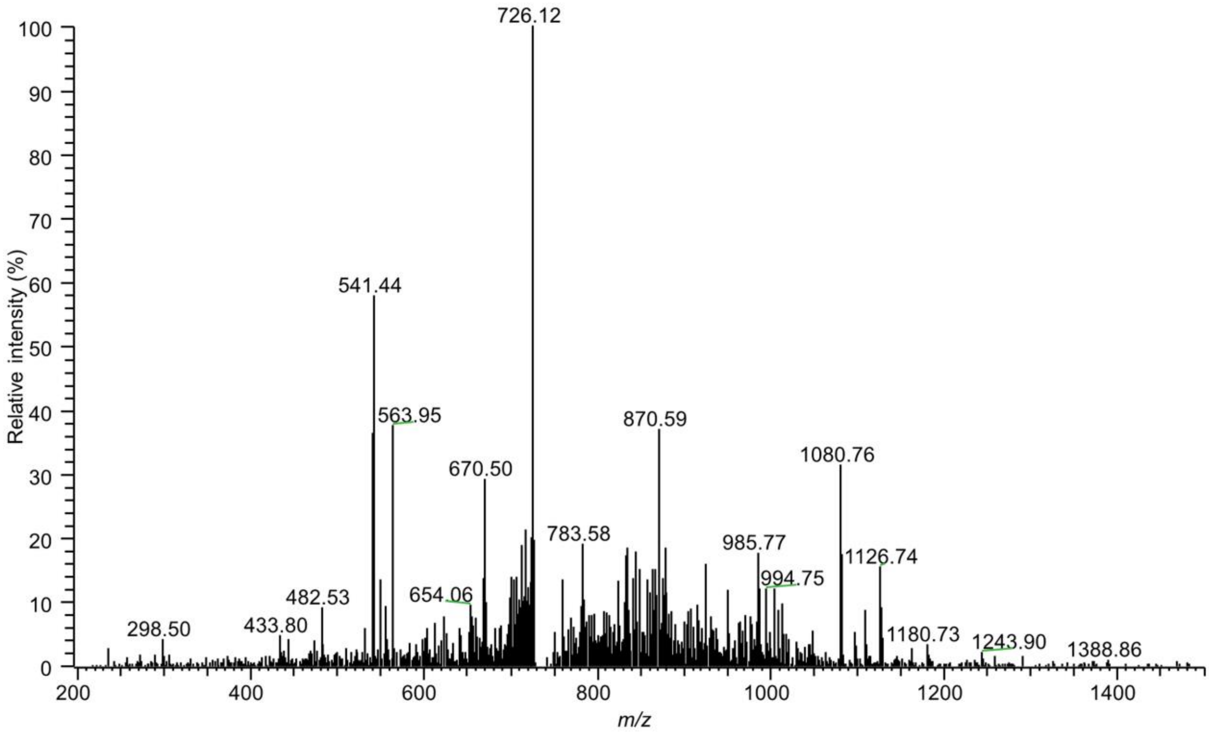

Figure S4-27 MS/MS spectrum of **VTFHNK$_{\mathbf{Am}}$GAYPLSIEPIGVR**

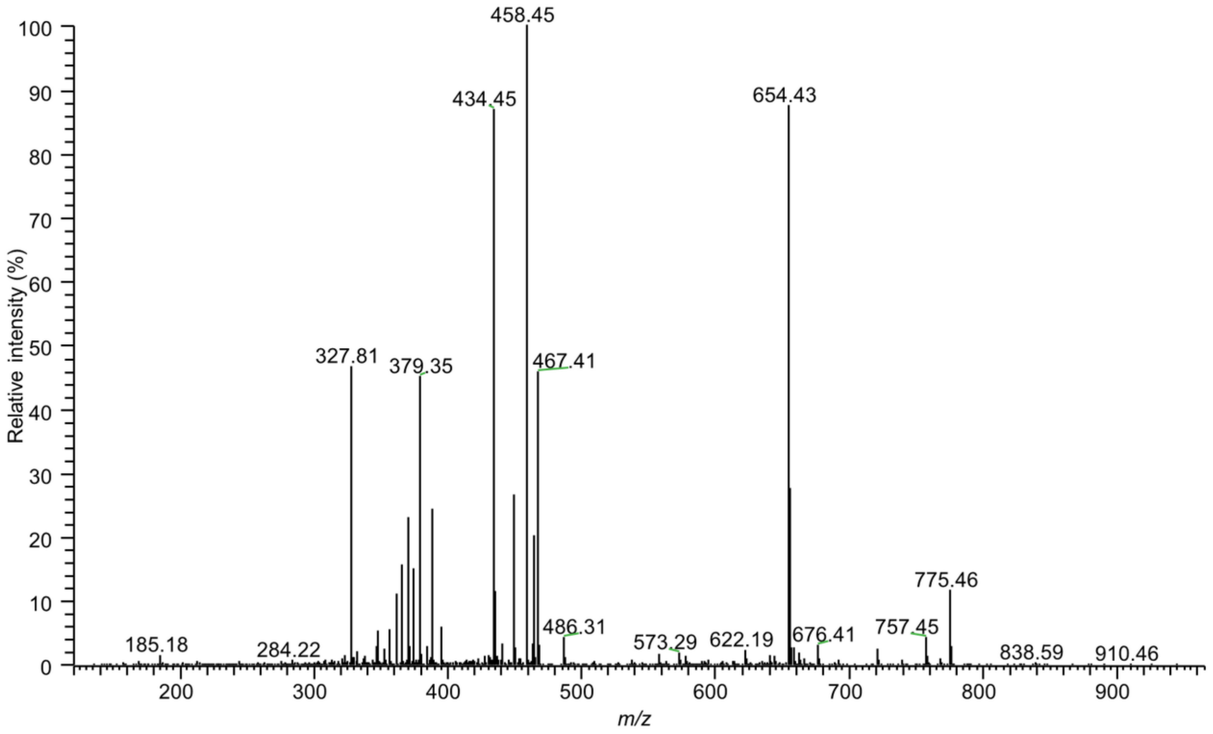

Figure S4-28 MS/MS spectrum of **VSNKAmALPAPIEK**

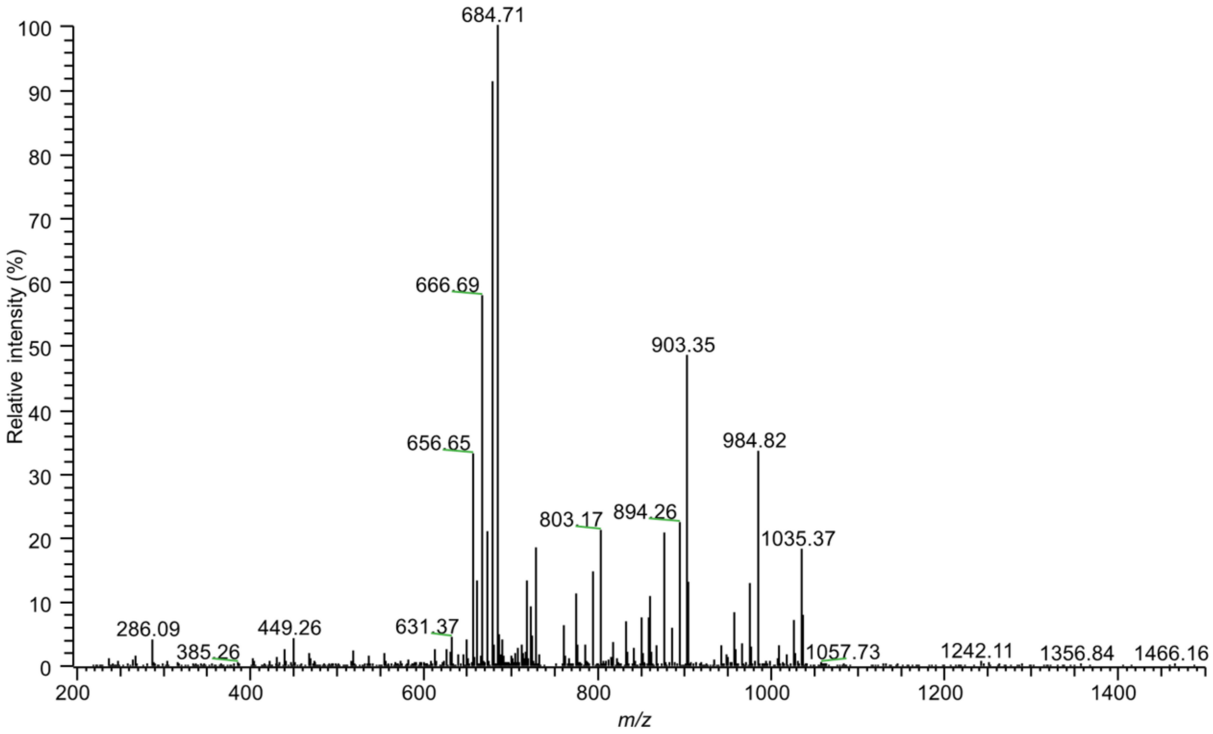

Figure S4-29 MS/MS spectrum of **DSTYSLSSTLTLSKAmADYEK**

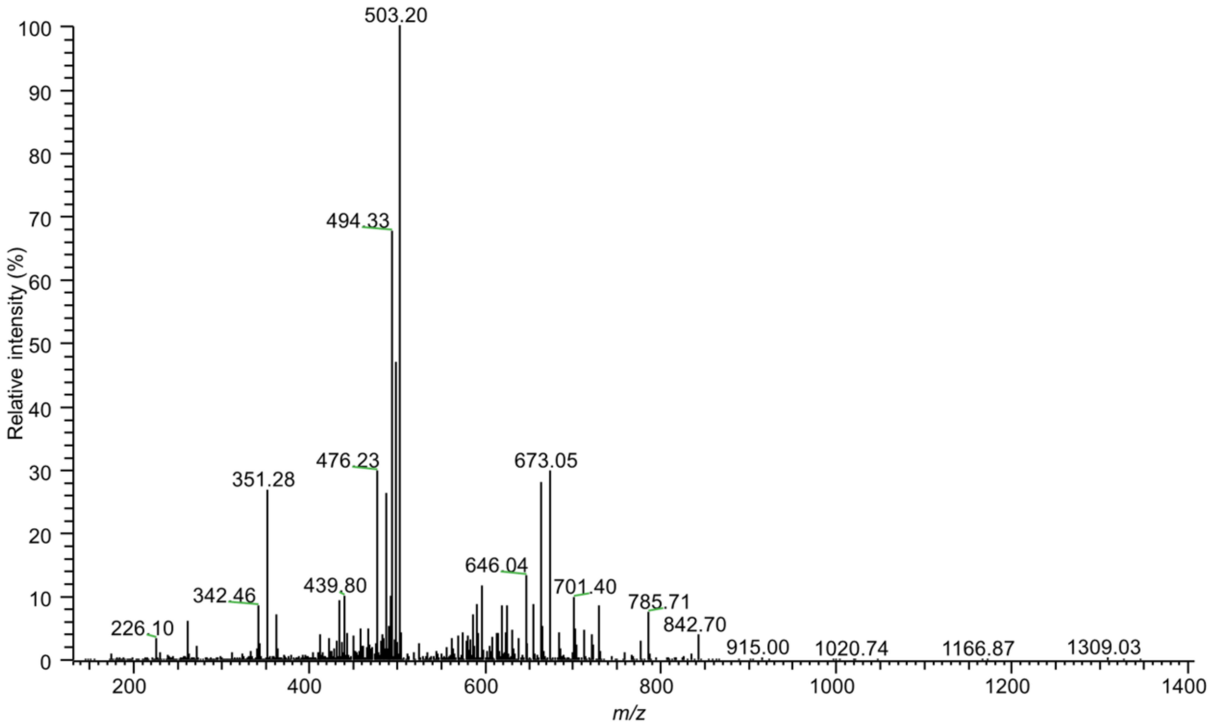

Figure S4-30 MS/MS spectrum of **GDVAFVK$_{Am}$HQTVPQNTGGK**

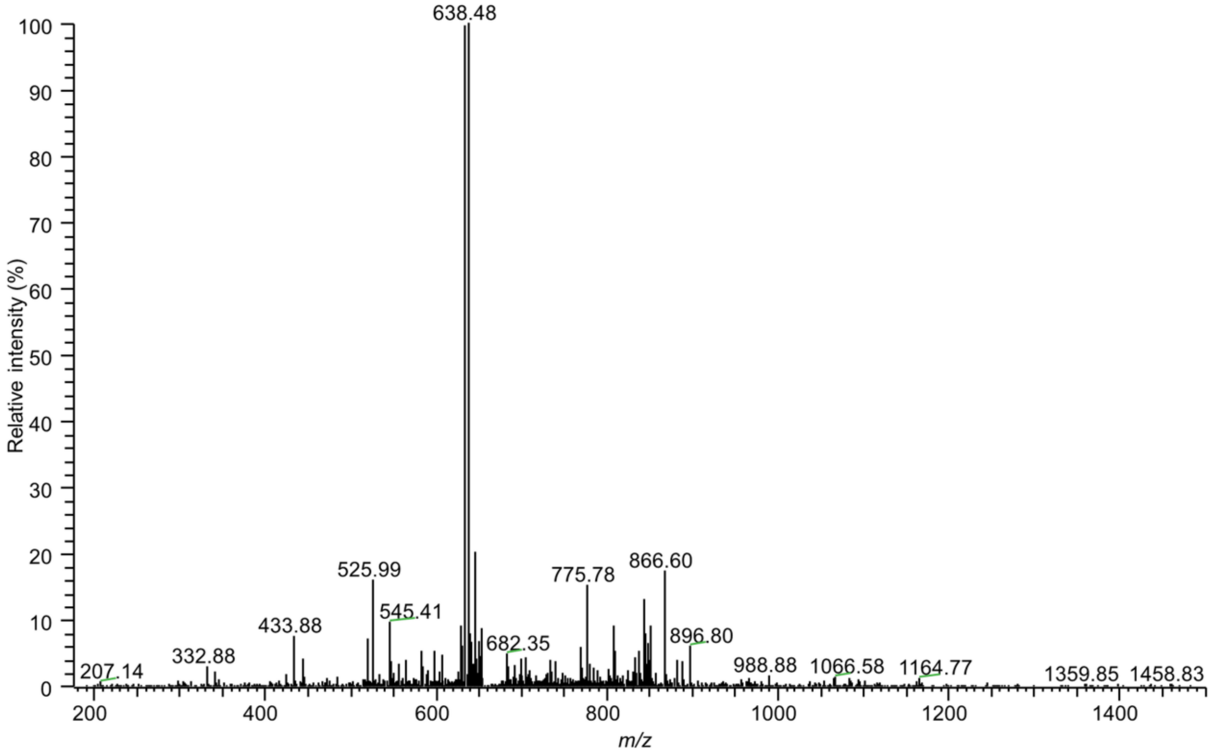

Figure S4-31 MS/MS spectrum of **K$_{Am}$PVDEYKDC$_{CAM}$HLAQVPSHTVVAR**

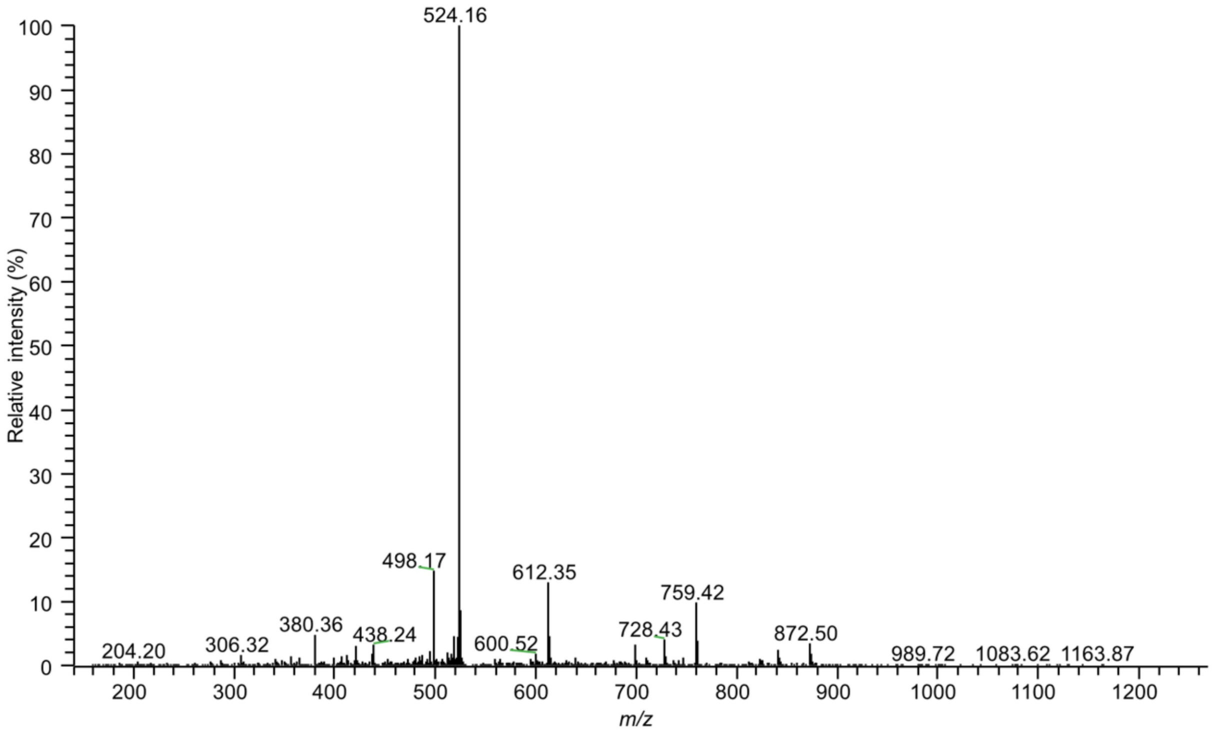

Figure S4-32 MS/MS spectrum of **SK_{Am}EFQLFSSPHGK**

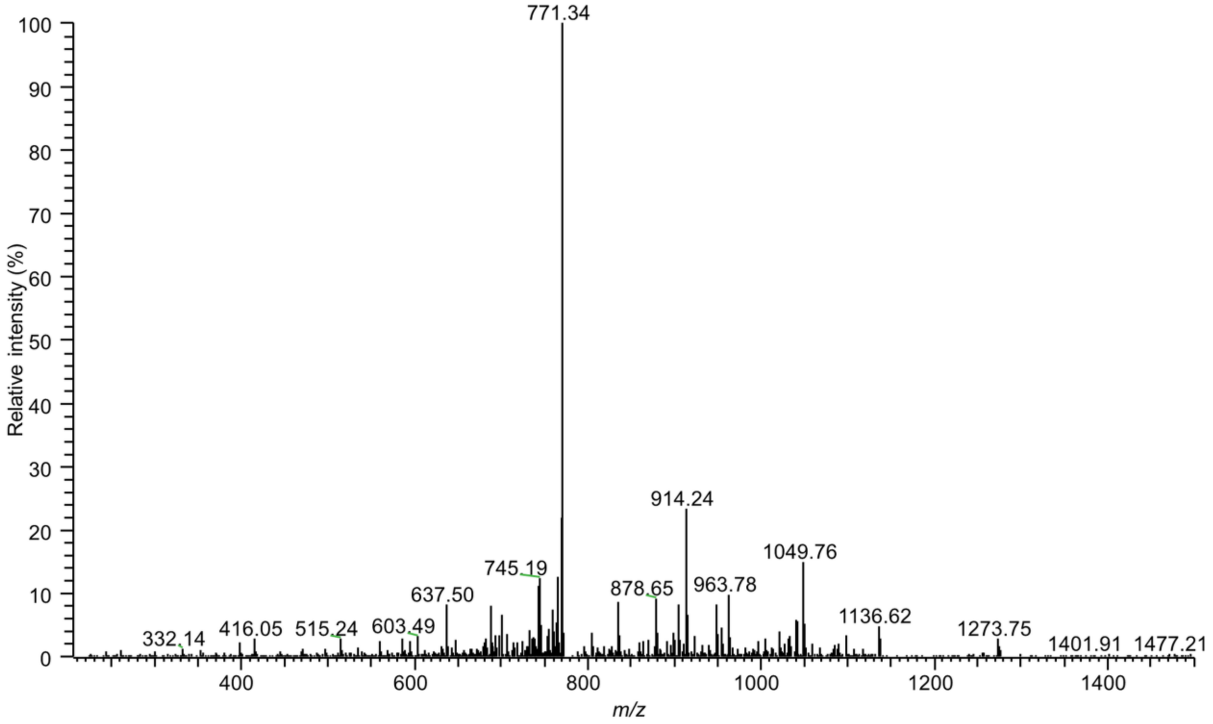

Figure S4-33 MS/MS spectrum of **DGAGDVAFVK_{Am}HSTIFENLANK**

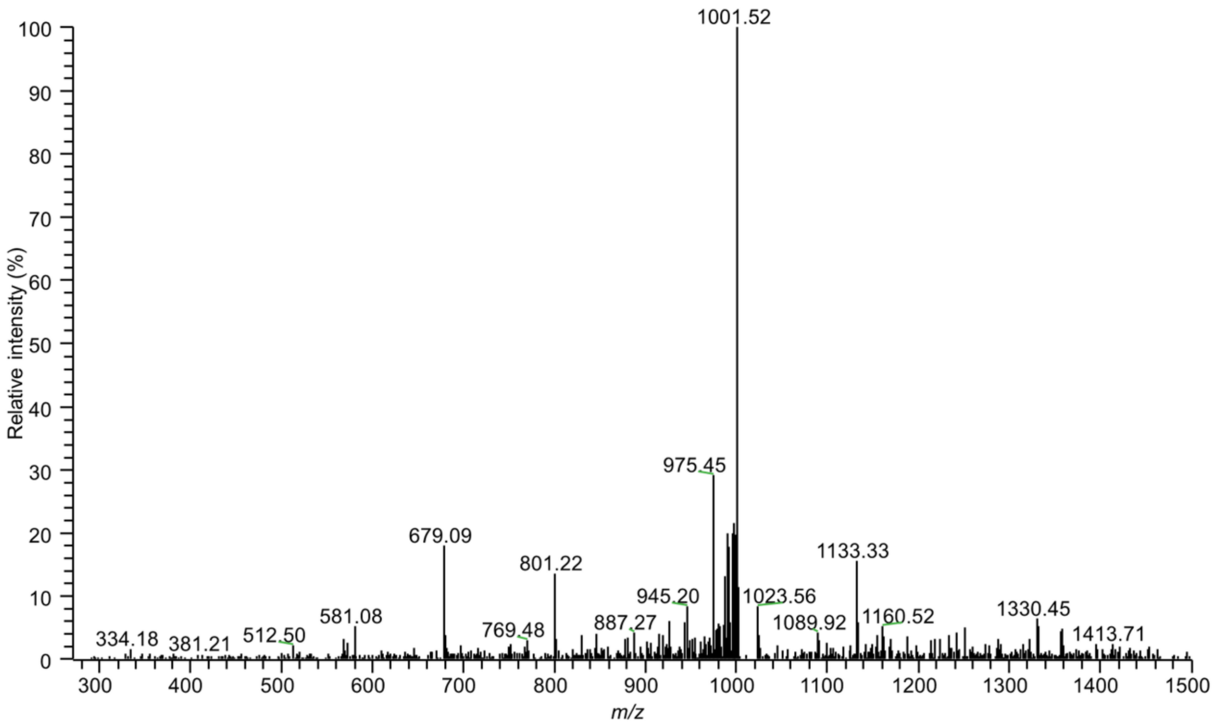

Figure S4-34 MS/MS spectrum of

TSALSAK$_{\mathbf{Am}}$**SC**$_{\mathbf{CAM}}$**ESNSPFPVHPGTAEC**$_{\mathbf{CAM}}$**C**$_{\mathbf{CAM}}$**TK**

Figure S5 Tandem mass spectra of differentially glycated peptides identified solely by manual interpretation of the MS/MS data. K_{Am} and C_{CAM} denote glycated lysyl and carbamidomethylated cysteinyl residues, respectively; * means fragment ions displayed losses of 54, 84 and 96 u characteristic for D-fructose-derived ions.

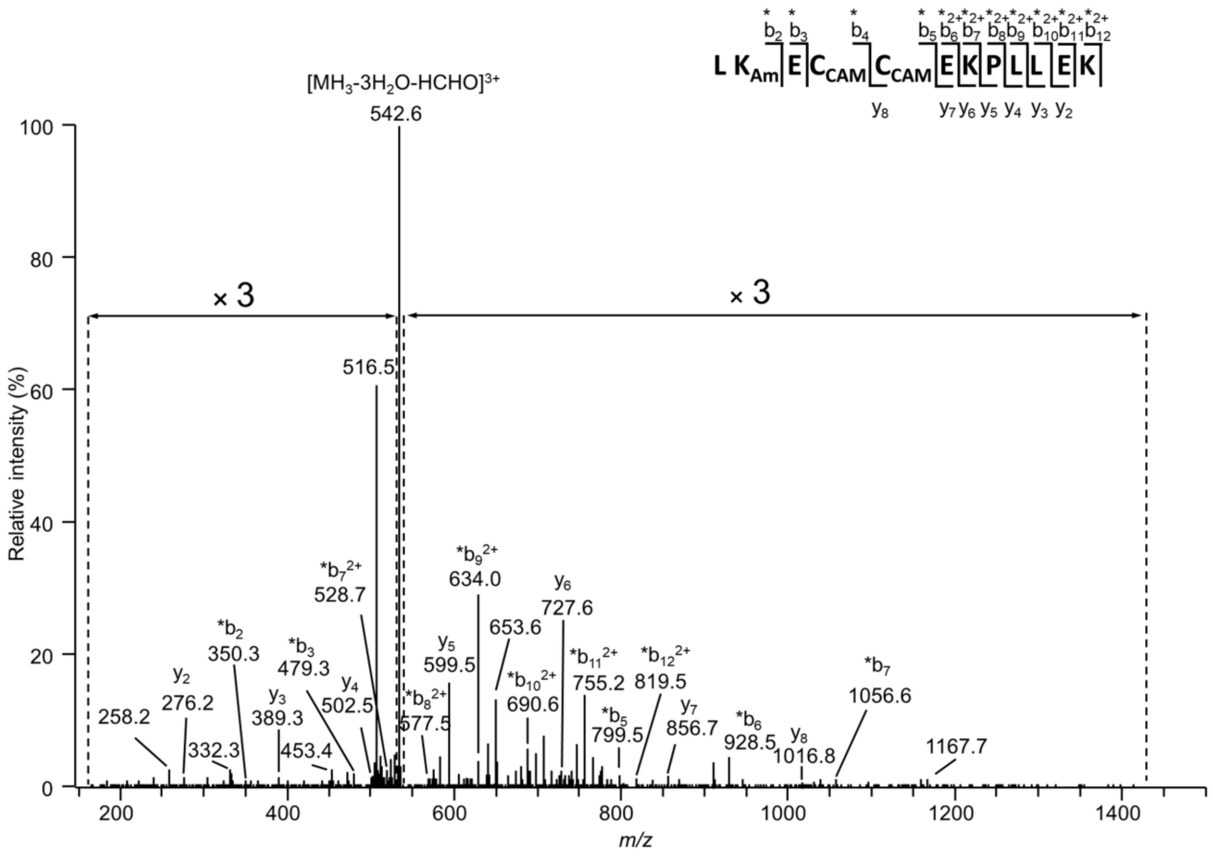

Figure S5-1 MS/MS spectrum of LKAmECCAMCCAMEKPLLEK.

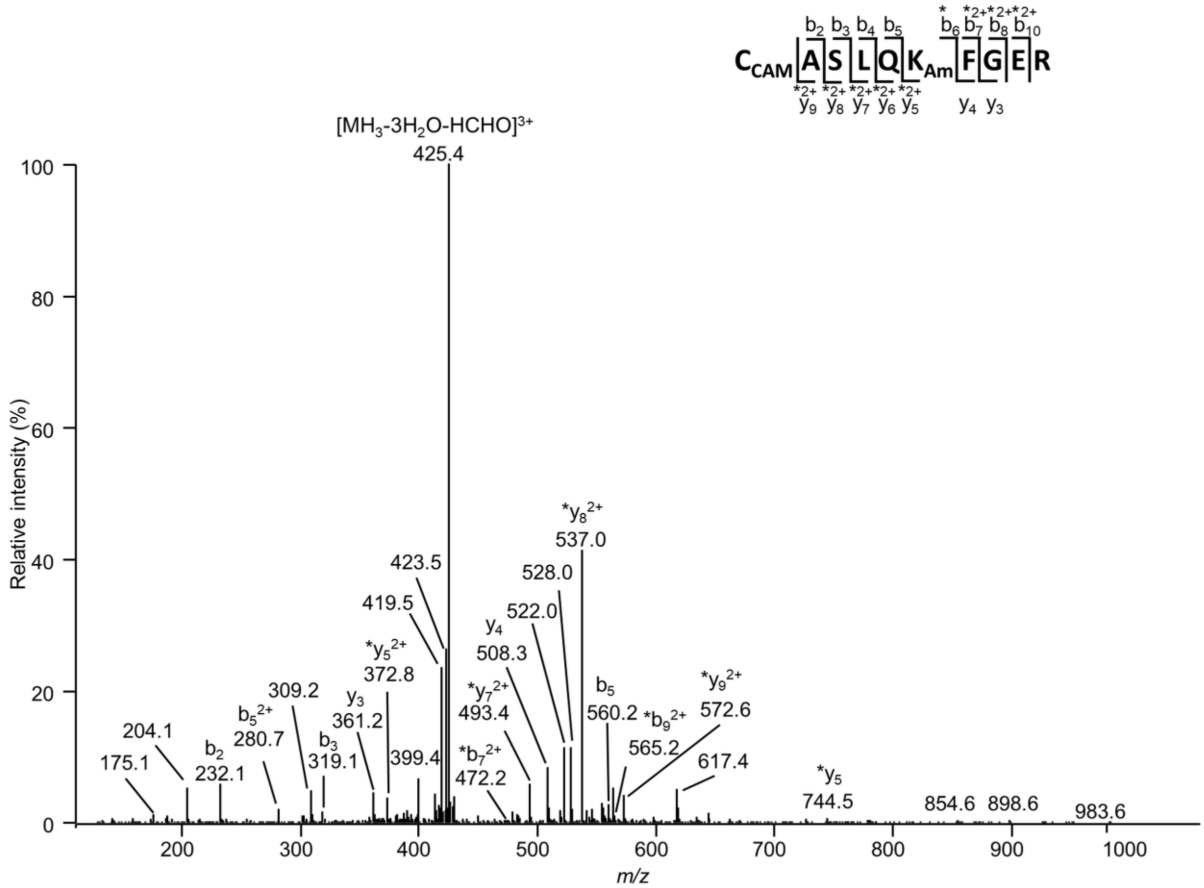

Figure S5-2 MS/MS spectrum of CCAMASLQKAmFGER.

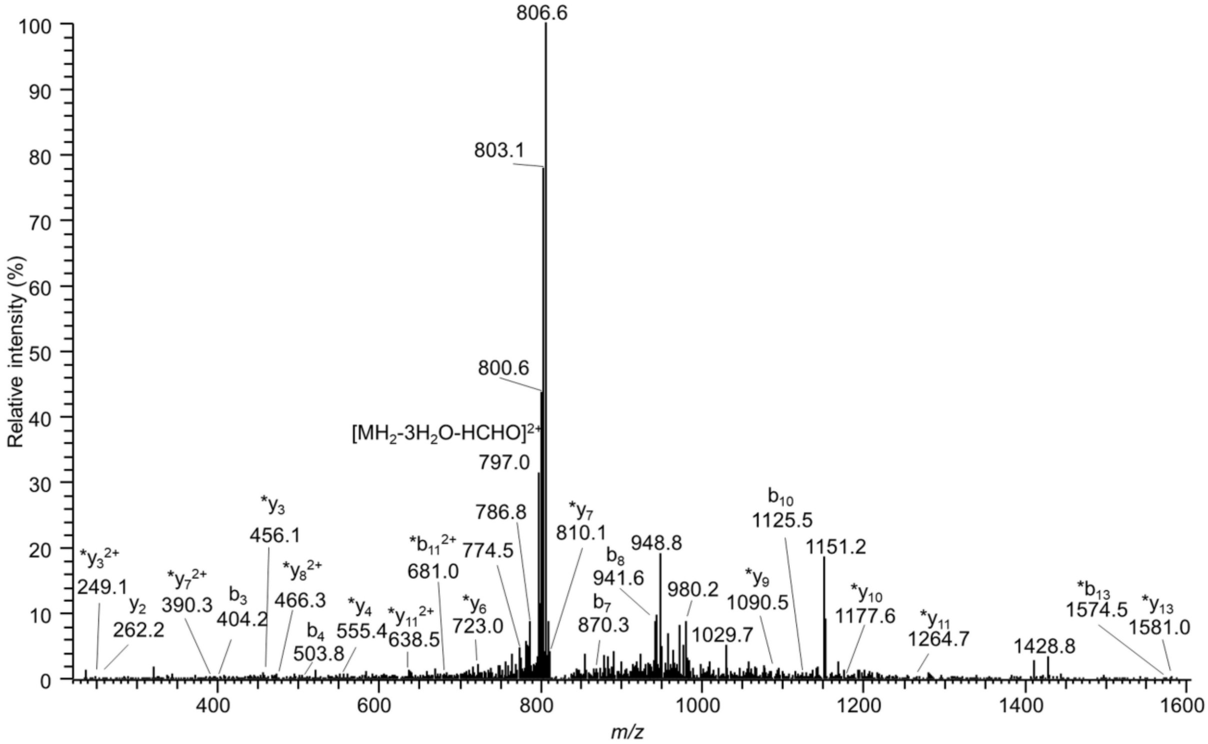

Figure S5-3 MS/MS spectrum of **RC$_{CAM}$SVFYGAPSK$_{Am}$SR**

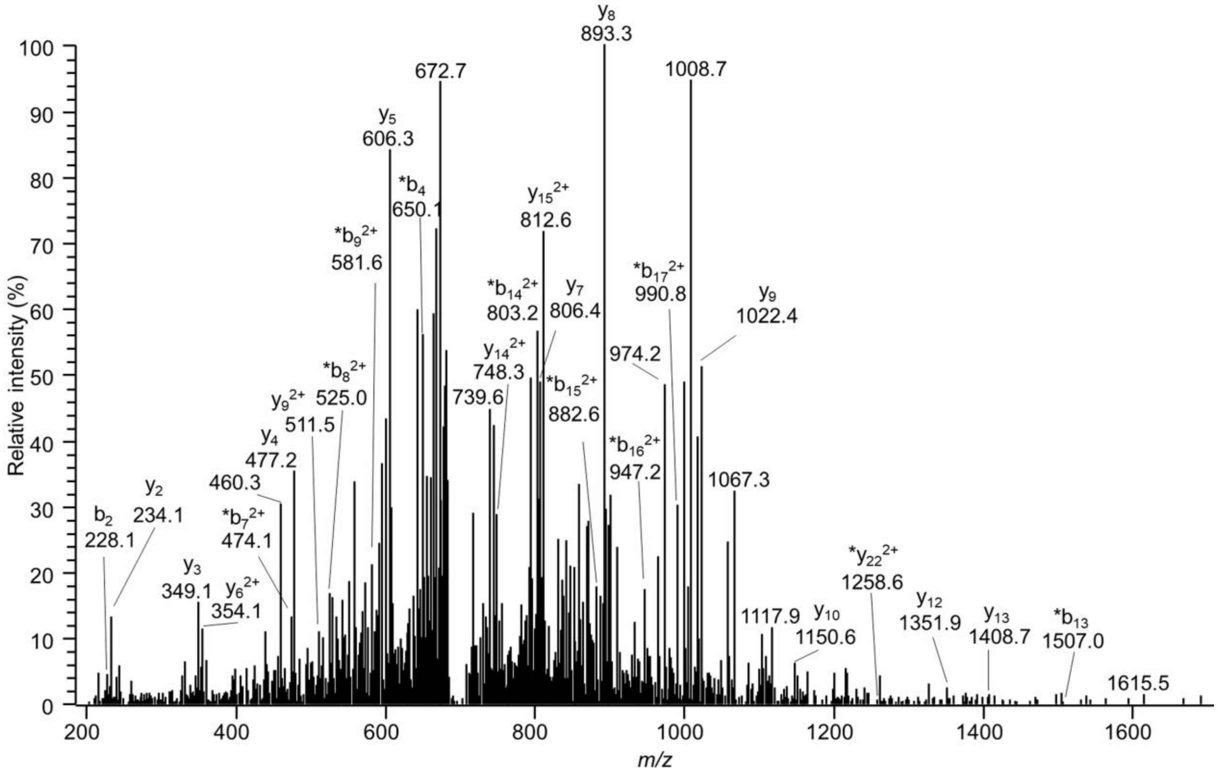

Figure S5-4 MS/MS spectrum of **VQWK$_{Am}$VDNALQSGNSQESVTEQDSK**

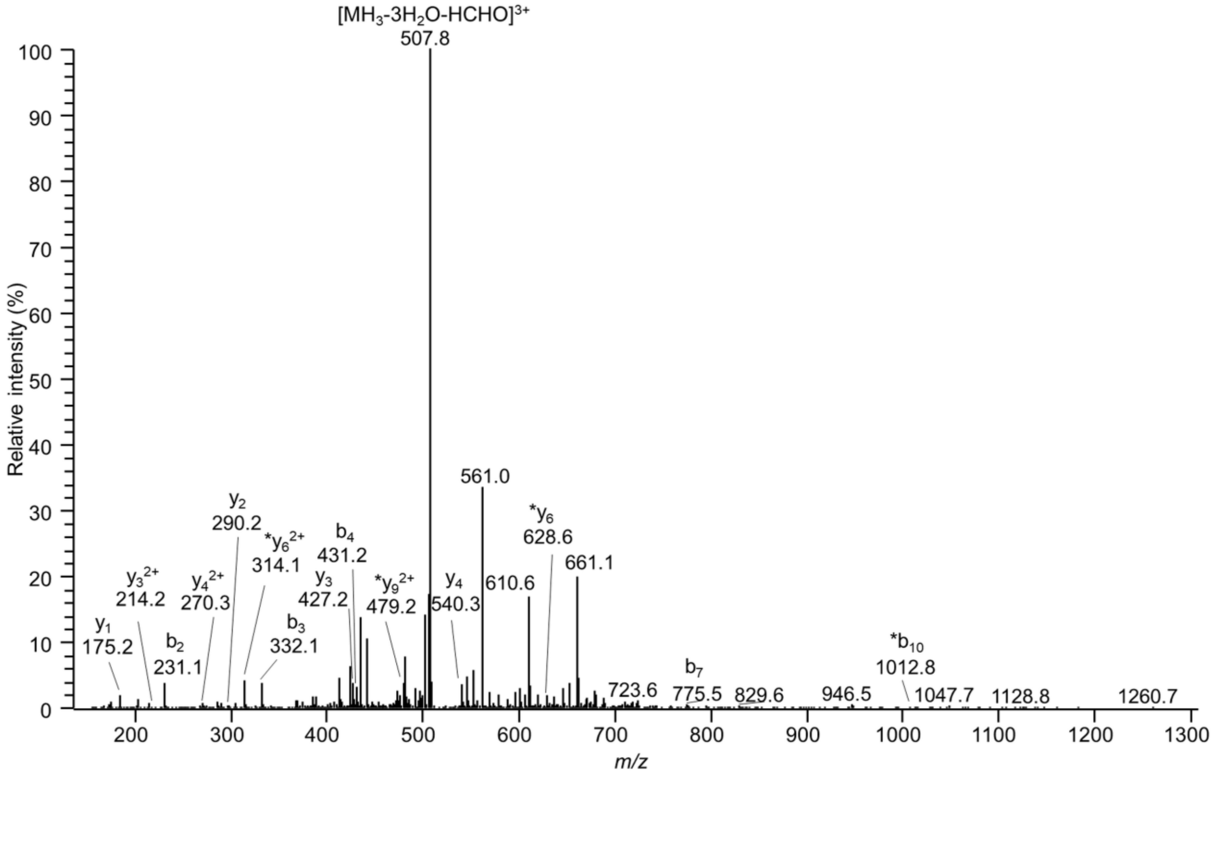

Figure S5-5 MS/MS spectrum of **DDTVC**$_{\mathbf{CAM}}$**LAK**$_{\mathbf{Am}}$**LHDR**

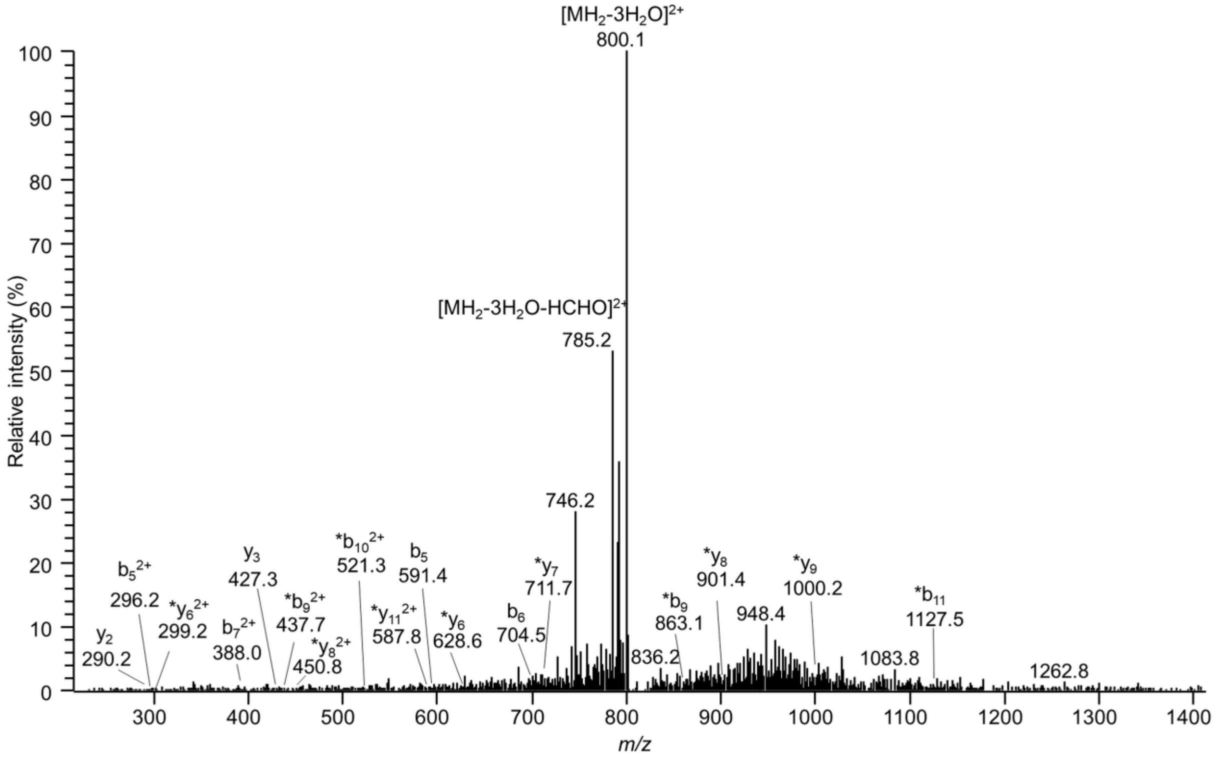

Figure S5-6 MS/MS spectrum of **DLLFK**$_{\mathbf{Am}}$**DSAHGFLK**

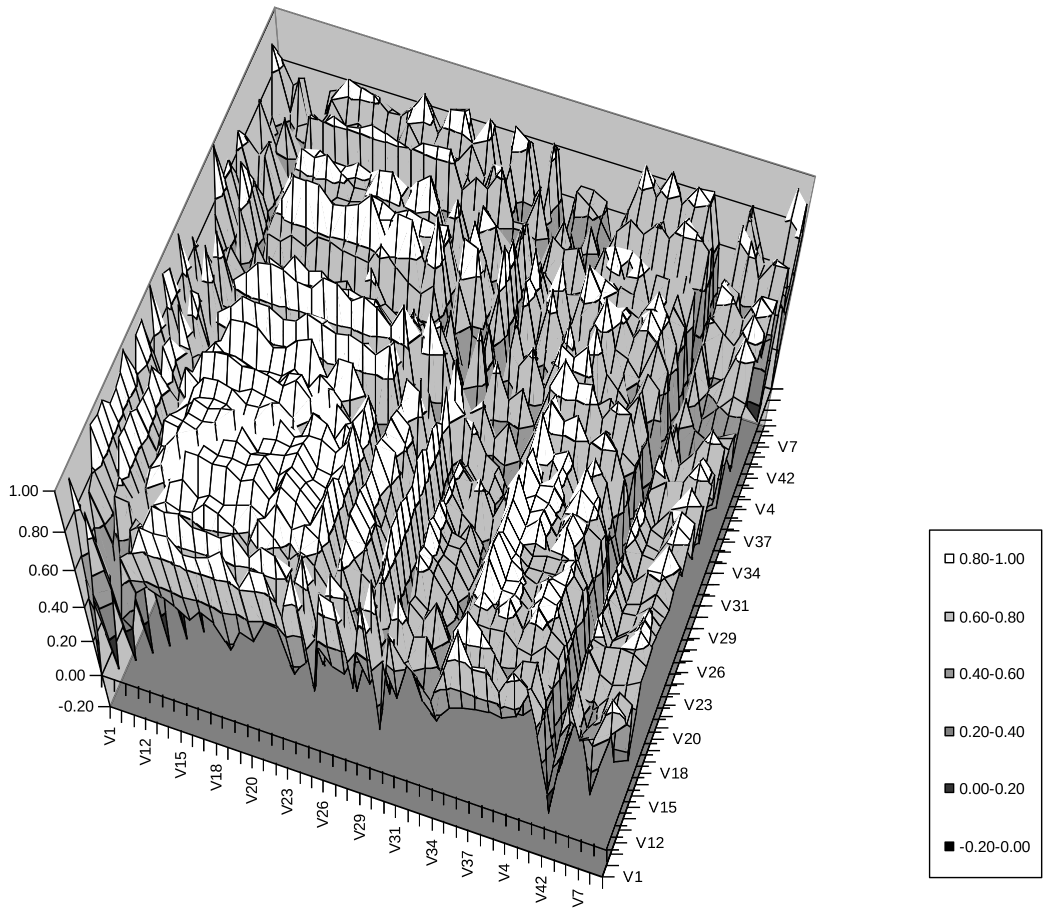

Figure S6 Pearson's cross-correlation coefficient plot for variables
